# Supplementary material for: Layered feedback control overcomes performance trade-off in synthetic biomolecular networks
Source: Nat Commun. 2022 Sep 14;13:5393. doi: 10.1038/s41467-022-33058-6 (PMC9474519; doi:10.1038/s41467-022-33058-6)
Supplement: Supplementary file 1 — Supplementary Information [file 41467_2022_33058_MOESM1_ESM.pdf]

Layered Feedback Control Overcomes Performance Trade-off in Synthetic Biomolecular Networks
Supplementary Information

Chelsea Y. Hu[1,2] and Richard M. Murray[1]

[1]Division of Biology and Biological Engineering, California Institute of Technology, Pasadena, CA.
[2]Department of Chemical Engineering, Texas A&M University, College Station, TX.

Contents

1 Supplementary Dynamical Models **2**
1.1 Reduced biomolecular model with coupled dynamics of gene expression and cell growth . . 2
1.1.1 Equations 2
1.1.2 Model species and parameters 3
1.2 Reduced biomolecular model with sRNA regulator folding delay 4
1.2.1 Equations 4
1.2.2 Model species and parameters 5
1.3 Reduced biomolecular model with translational capping 6
1.3.1 Equations 6
1.3.2 Model species and parameters 6

2 Supplementary Experimental Results **7**
2.1 Construct functionality requires RBS optimization 7
2.2 Growth profile of the synthetic network-hosting cells 8

3 Supplementary Simulation Results **8**
3.1 Impulse response analysis using a linearized state space model 8
3.2 Simulated dynamics using generic biomolecular model with parameter randomization 9

4 Dynamical Perturbation Experiments **10**
4.1 Data exclusion procedures based on growth profile 10
4.2 System dynamics under various types of perturbations 11
4.3 Detailed perturbation experiment protocols 12
4.4 Supplementary Statistical Results 12

5 Parameter estimates **15**

1 Supplementary Dynamical Models

1.1 Reduced biomolecular model with coupled dynamics of gene expression and cell growth

To model the biomolecular dynamics coupled with cell growth, we wrote the following equations of 15 species and 25 parameters. Species 1-14 are in the single cell scale. The last species C is in the populational scale.

1.1.1 Equations

$$\frac{dM_{cin}}{dt} = \boldsymbol{\beta_{rhl}} \cdot (\frac{X_{rhl}}{X_{rhl}+K_{rhl}} + l_{rhl}) \cdot f_{trans} - \boldsymbol{d_m} \cdot M_{cin} + k_r \cdot T_{cin} - M_{cin} \cdot B \cdot k_{tf} \quad (1.1)$$

$$\frac{dP_{cin}}{dt} = k_r \cdot T_{cin} - m_r \cdot P_{cin} - \boldsymbol{d_g} \cdot P_{cin} - \boldsymbol{d_p} \cdot P_{cin} \quad (1.2)$$

$$\frac{dC_{ind}}{dt} = m_r \cdot P_{cin} - \boldsymbol{d_g} \cdot C_{ind} - \boldsymbol{d_p} \cdot C_{ind} \quad (1.3)$$

$$\frac{dR}{dt} = \boldsymbol{\beta_{cin}} \cdot (\frac{C_{ind}}{C_{ind}+K_{cin}} + l_{cin}) \cdot f_{cis} - \boldsymbol{d_r} \cdot R - m_{as} \cdot R \quad (1.4)$$

$$\frac{dM}{dt} = \boldsymbol{\beta_{cin}} \cdot (\frac{C_{ind}}{C_{ind}+K_{cin}} + l_{cin}) \cdot f_{cis} - \boldsymbol{d_m} \cdot M + k_r \cdot T_{lac} - M \cdot B \cdot k_{tf} + T_{lac} \cdot k_{lac_R} + k_r \cdot T_{fp} - M \cdot B \cdot k_{tf} + T_{fp} \cdot k_{fp_R} \quad (1.5)$$

$$\frac{dP_{lac}}{dt} = k_r \cdot T_{lac} - \boldsymbol{d_g} \cdot P_{lac} - \boldsymbol{d_p} \cdot P_{lac} - m_{lac} \cdot P_{lac} \quad (1.6)$$

$$\frac{dB}{dt} = -M_{cin} \cdot B \cdot k_{tf} + T_{cin} \cdot k_{cin_R} - 2 \cdot M \cdot B \cdot k_{tf} + T_{lac} \cdot k_{lac_R} + T_{fp} \cdot k_{fp_R} + k_r \cdot (T_{cin} + T_{lac} + T_{fp}) - \boldsymbol{d_p} \cdot B \quad (1.7)$$

$$\frac{dP_{fp}}{dt} = k_r \cdot T_{fp} - \alpha \cdot P_{fp} - \boldsymbol{d_g} \cdot P_{fp} - \boldsymbol{d_p} \cdot P_{fp} \quad (1.8)$$

$$\frac{dT_{cin}}{dt} = -k_r \cdot T_{cin} + M_{cin} \cdot B \cdot k_{tf} - T_{cin} \cdot k_{cin_R} - \boldsymbol{d_g} \cdot T_{cin} \quad (1.9)$$

$$\frac{dT_{lac}}{dt} = -k_r \cdot T_{lac} + M \cdot B \cdot k_{tf} - T_{lac} \cdot k_{lac_R} - \boldsymbol{d_g} \cdot T_{lac} \quad (1.10)$$

$$\frac{dT_{fp}}{dt} = -k_r \cdot T_{fp} + M \cdot B \cdot k_{tf} - T_{fp} \cdot k_{fp_R} - \boldsymbol{d_g} \cdot T_{fp} \quad (1.11)$$

$$\frac{dR_m}{dt} = m_{as} \cdot R - \boldsymbol{d_r} \cdot R_m \quad (1.12)$$

$$\frac{dP_{m_{lac}}}{dt} = m_{lac} \cdot P_{lac} - \boldsymbol{d_g} \cdot P_{m_{lac}} - \boldsymbol{d_p} \cdot P_{m_{lac}} \quad (1.13)$$

$$\frac{dP_{fp}}{dt} = \alpha \cdot P_{fp} - \boldsymbol{d_g} \cdot P_{m_{fp}} - \boldsymbol{d_p} \cdot P_{fp} \quad (1.14)$$

$$\frac{dC}{dt} = r_g \cdot (1 - \frac{C}{C_{max}}) \cdot C \quad (1.15)$$

Note that the terms in red ink describes the translational mechanisms of the three protein species: CinR, lacI, and sfYFP. The chemical reactions are described by the following chemical equation:

$$Ribosome(B) + mRNA(M_x) \underset{k_{xR}}{\overset{k_{tf}}{\rightleftharpoons}} Transcript(T_x) \xrightarrow{k_r} Protein(P_x)$$

Also, to connect gene expression to resource limitation, we wrote the bolded parameters as functions of growth and total species in a cell. Specifically, proteins are diluted (d_g) by cell division, which slows down when population reaches density capacity. At the same time, the degradation rates of proteins (d_p) are also functions of growth, which speeds up when cell population reaches density capacity as the resources are limited and dead cells become the main resource of amino acids in the growth medium; and they also slow down when there is an abundance of total proteins (P_{tot}). The transcriptional rates β_{rhl} and β_{cin} are also modeled as functions of cell population and species population. Finally, we modeled the degradation rate of RNA species as a function of total RNA in the cell (R_{tot}).

$$
\begin{aligned}
d_g &= r_g \cdot (1 - \frac{C}{C_{max}}) && (1.16) \\
d_p &= d_{p_{max}} \cdot (\frac{C}{C_{max}}) \cdot (1 - \frac{P_{tot}}{P_{max}}) && (1.17) \\
\beta_{rhl} &= \beta_{rhl_{max}} \cdot (1 - \frac{R_{tot}}{R_{max}}) \cdot (1 - \frac{C}{C_{max}}) && (1.18) \\
\beta_{cin} &= \beta_{cin_{max}} \cdot (1 - \frac{R_{tot}}{R_{max}}) \cdot (1 - \frac{C}{C_{max}}) && (1.19) \\
d_r &= d_{r_{max}} \cdot (1 - \frac{R_{tot}}{R_{max}}) && (1.20) \\
d_m &= d_{m_{max}} \cdot (1 - \frac{R_{tot}}{R_{max}}) && (1.21) \\
R_{tot} &= M_{cin} + R + M + T_{cin} + T_{lac} + T_{fp} + R_m && (1.22) \\
P_{tot} &= P_{cin} + C_{ind} + P_{lac} + B + P_{fp} + P_{m_{lac}} + P_{fp} && (1.23)
\end{aligned}
$$

Additionally, f_{cis} and f_{trans} denote for the *cis* and the *trans* feedbacks in the system.
For the open loop: $f_{cis} = 1; f_{trans} = 1$
For the *cis* feedback only: $f_{cis} = \frac{K_R}{K_R+R_m}; f_{trans} = 1$
For the *trans* feedback only: $f_{cis} = 1; f_{trans} = \frac{K_{lac}}{K_{lac}+P_{m_{lac}}}$
For the layered feedback: $f_{cis} = \frac{K_R}{K_R+R_m}; f_{trans} = \frac{K_{lac}}{K_{lac}+P_{m_{lac}}}$

1.1.2 Model species and parameters

Supplementary Table 1: Growth Dependent Biomolecular Model Species

Species	Description
M_{ind}	mRNA of CinR
P_{ind}	CinR translated peptides
C_{ind}	CinR activating complex, with folded CinR bonded with AHL-Cin molecules
R	antisense sRNA repressor unfolded
M	mRNA containing sRNA, sfYFP and LacI
P_{lac}	LacI protein translated peptides
P_{fp}	sfYFP protein translated peptides
B	Ribosome
T_{cin}	Ribosome bound CinR mRNA
T_{lac}	Ribosome bound LacI mRNA
T_{fp}	Ribosome bound sfYFP mRNA
R_m	folded mature antisense sRNA
$P_{m_{lac}}$	folded mature LacI protein
$P_{m_{fp}}$	folded mature sfYFP
C	Number of cells in the culture

Supplementary Table 2: Growth Dependent Biomolecular Model Parameters

Parameters	Description	Unit	Estimates
$\beta_{rhl_{max}}$	Max transcription rate of inducible promoter $P_{rhl/LacO}$	fM/min	5
K_{rhl}	Activation coefficient of inducer Rhl	nM	1.4e5
K_R	Repression coefficient of the sRNA repressor	fM	20
$d_{m_{max}}$	Max degradation/dilution rate of mRNA	1/min	0.2
k_{cin_R}	Unbinding rate of ribosome B and CinR mRNA	1/min	3000
l_{rhl}	Leak coefficient of $P_{rhl/LacO}$ promoter	N/A	0.10
m_r	Maturation rate of CinR-AHL complex	1/min	0.05
K_{cin}	Activation coefficient of P_{cin}	fM	80
$\beta_{cin_{max}}$	Max transcription rate of P_{cin} promoter inducible by C_{ind}	fM/min	20
$d_{r_{max}}$	Max degradation/dilution rate of sRNA	1/min	0.15
m_{fp}	Maturation rate of sfYFP	1/min	0.01
x_{rhl}	The activating inducer for $P_{rhl/LacO}$	nM	3e5
k_{fp_R}	Unbinding rate of ribosome B and sfYFP mRNA	1/min	1000
k_{tf}	Forward binding rate of mRNA and ribosome	1/min	1
K_r	Translate and release rate of activated transcript	1/min	0.015
k_{lac_R}	Unbinding rate of ribosome B and LacI mRNA	1/min	3000
K_{lac}	Repression coefficient of LacI	fM	30
$d_{p_{max}}$	Max protein degradation rate	1/min	0.007
r_g	Max growth rate of cells	1/min	0.012
C_{max}	Population capacity	count	8e8
m_{AS}	Maturation rate of sRNA	1/min	0.02
m_{lac}	Maturation rate of LacI protein	1/min	0.05
R_{max}	RNA capacity in a cell	fM	10000
P_{max}	Protein capacity in a cell	fM	10000
l_{cin}	Leak coefficient of P$_{cin}$	N/A	0.1

1.2 Reduced biomolecular model with sRNA regulator folding delay

1.2.1 Equations

$$\frac{dM_{ind}}{dt} = f_{tx} \cdot \beta_A \cdot (\frac{x}{K_x + x}) \cdot f_{trans} - d_m \cdot M_{ind} \quad (1.24)$$
$$\frac{dP_{ind}}{dt} = f_{tl} \cdot k_{tp} \cdot M_{ind} - d \cdot P_{ind} - K_r \cdot P_{ind} \quad (1.25)$$
$$\frac{dC_{ind}}{dt} = K_r \cdot P_{ind} - d \cdot C_{ind} \quad (1.26)$$
$$\frac{dM_G}{dt} = f_{tx} \cdot \beta_B \cdot (\frac{C_{ind}}{K_{ind} + C_{ind}}) \cdot f_{cis} - d_m \cdot M_G \quad (1.27)$$
$$\frac{dR}{dt} = f_{tx} \cdot \beta_B \cdot (\frac{C_{ind}}{K_{ind} + C_{ind}}) \cdot f_{cis} - d_r \cdot R - \alpha_r \cdot R \quad (1.28)$$
$$\frac{dR_f}{dt} = \alpha_r \cdot R - d_r \cdot R_f \quad (1.29)$$
$$\frac{dP}{dt} = f_{tl} \cdot k_{tr} \cdot M_G - d \cdot P \quad (1.30)$$
$$\frac{dG}{dt} = f_{tl} \cdot k_{tg} \cdot M_G - \alpha \cdot G - d \cdot G \quad (1.31)$$
$$\frac{dG_m}{dt} = \alpha \cdot G - d \cdot G_m \quad (1.32)$$

If transcription is regulated by a protein species, the Hill function is written as $f_{Hill_P} = \frac{K_p}{K_p+P}$. If transcription is regulated by a sRNA species, the Hill function is written as $f_{Hill_R} = \frac{K_R}{K_R+R_f}$. For the open

loop, $f_{cis} = f_{trans} = 1$. For the *trans* only feedback, $f_{cis} = 1$ and $f_{trans} = f_{Hill_P}$. For the *cis* feedback, $f_{trans} = 1$ and $f_{cis} = f_{Hill_R}$. For the layered feedbacks, $f_{cis} = f_{Hill_R}, f_{trans} = f_{Hill_P}$.

1.2.2 Model species and parameters

Supplementary Table 3: Species Table for Generic Biomolecular Model with sRNA Folding Delay

Species	Description
M_{ind}	the mRNA of signaling protein P_{ind}
P_{ind}	the signaling protein translated peptides
C_{ind}	the signaling complex, with folded signaling protein bound with inducer molecules
R	unfolded the regulator sRNA repressor
R_f	properly folded sRNA regulator repressor
P	the regulator protein repressor
M_G	the GOI mRNA transcript
G	the translated GOI peptides
G_m	the mature GOI (observable)

Supplementary Table 4: Parameter Table for Generic Biomolecular Model with sRNA Folding Delay

Parameters	Description	Unit	Estimates
β_A	max transcription rate of the inducible promoter P_x	fM/min	2
K_x	activation coefficient of the chemical inducer x	nM	1.4e4
K_R	repression coefficient of the sRNA repressor	fM	20
K_p	repression coefficient of the protein repressor	fM	200
d_m	degradation/dilution rate of mRNA	1/min	0.1
k_{tp}	translation rate of the inducing protein P_{ind}	1/min	0.1
d	degradation/dilution rate of all proteins, dominated by dilution	1/min	0.03
K_r	the maturation rate of the activating complex	1/min	0.1
β_B	max transcription rate of the C_{ind} inducible promoter	fM/min	20
K_{ind}	activation coefficient of C_{ind}	nM	200
d_r	degradation/dilution rate of sRNA	1/min	0.3
α	maturation rate of GOI	1/min	0.2
x	the chemical inducer that activates P_x	nM	2.0e6
k_{tg}	translation rate of GOI	1/min	0.1
f_{tx}	scaling factor of universal transcription	NA	1
f_{tl}	scaling factor of universal translation	NA	1
k_{tr}	translation rate of the regulator protein	1/min	0.04
α_r	sRNA folding rate	1/min	0.5

1.3 Reduced biomolecular model with translational capping

1.3.1 Equations

$$\frac{dM_{ind}}{dt} = f_{tx} \cdot \beta_A \cdot (\frac{x}{K_x + x}) \cdot f_{trans} - d_m \cdot M_{ind} \tag{1.33}$$

$$\frac{dP_{ind}}{dt} = f_{tl} \cdot k_{tp} \cdot M_{ind} - d \cdot P_{ind} - K_r \cdot P_{ind} \tag{1.34}$$

$$\frac{dC_{ind}}{dt} = K_r \cdot P_{ind} - d \cdot C_{ind} \tag{1.35}$$

$$\frac{dM_G}{dt} = f_{tx} \cdot \beta_B \cdot (\frac{C_{ind}}{K_{ind} + C_{ind}}) \cdot f_{cis} - d_m \cdot M_G \tag{1.36}$$

$$\frac{dR}{dt} = f_{tx} \cdot \beta_B \cdot (\frac{C_{ind}}{K_{ind} + C_{ind}}) \cdot f_{cis} - d_r \cdot R \tag{1.37}$$

$$\frac{dP}{dt} = f_{tl} \cdot k_{tr} \cdot M_G - d \cdot P \tag{1.38}$$

$$\frac{dG}{dt} = f_{tl} \cdot k_{tg} \cdot M_G - \alpha \cdot G - d \cdot G \tag{1.39}$$

$$\frac{dG_m}{dt} = \alpha \cdot G - d \cdot G_m \tag{1.40}$$

$$R_{tot} = M_{ind} + M_G \tag{1.41}$$

$$k_{tp} = k_{tp-max} \cdot (1 - \frac{R_{tot}}{R_{max}}) \tag{1.42}$$

$$k_{tr} = k_{tr-max} \cdot (1 - \frac{R_{tot}}{R_{max}}) \tag{1.43}$$

$$k_{tg} = k_{tg-max} \cdot (1 - \frac{R_{tot}}{R_{max}}) \tag{1.44}$$

If transcription is regulated by a protein species, the Hill function is written as $f_{Hill_P} = \frac{K_p}{K_p+P}$. If transcription is regulated by a sRNA species, the Hill function is written as $f_{Hill_R} = \frac{K_R}{K_R+R}$. For the open loop, $f_{cis} = f_{trans} = 1$. For the *trans* only feedback, $f_{cis} = 1$ and $f_{trans} = f_{Hill_P}$. For the *cis* feedback, $f_{trans} = 1$ and $f_{cis} = f_{Hill_R}$. For the layered feedbacks, $f_{cis} = f_{Hill_R}, f_{trans} = f_{Hill_P}$.

1.3.2 Model species and parameters

Supplementary Table 5: Species Table for Generic Biomolecular Model with Translational Capping

Species	Description
M_{ind}	the mRNA of signaling protein P_{ind}
P_{ind}	the signaling protein translated peptides
C_{ind}	the signaling complex, with folded signaling protein bound with inducer molecules
R	unfolded the regulator sRNA repressor
P	the regulator protein repressor
M_G	the GOI mRNA transcript
G	the translated GOI peptides
G_m	the mature GOI (observable)

Supplementary Table 6: Parameter Table for Generic Biomolecular Model with Translational Capping

Parameters	Description	Unit	Estimates
β_A	max transcription rate of the inducible promoter P_x	fM/min	2
K_x	activation coefficient of the chemical inducer x	nM	1.4e4
K_R	repression coefficient of the sRNA repressor	fM	20
K_p	repression coefficient of the protein repressor	fM	200
d_m	degradation/dilution rate of mRNA	1/min	0.1
k_{tp-max}	max translation rate of the inducing protein P_{ind}	1/min	0.1
d	degradation/dilution rate of all proteins, dominated by dilution	1/min	0.03
K_r	the maturation rate of the activating complex	1/min	0.1
β_B	max transcription rate of the C_{ind} inducible promoter	fM/min	20/ 50
K_{ind}	activation coefficient of C_{ind}	nM	200
d_r	degradation/dilution rate of sRNA	1/min	0.3
α	maturation rate of GOI	1/min	0.2
x	the chemical inducer that activates P_x	nM	2.0e6
k_{tg-max}	max translation rate of GOI	1/min	0.5
f_{tx}	scaling factor of universal transcription	NA	1
f_{tl}	scaling factor of universal translation	NA	1
k_{tr-max}	max translation rate of the regulator protein	1/min	0.04/ 0.1
R_{max}	max mRNA used to cap translation	fM	500/ 800

2 Supplementary Experimental Results

2.1 Construct functionality requires RBS optimization

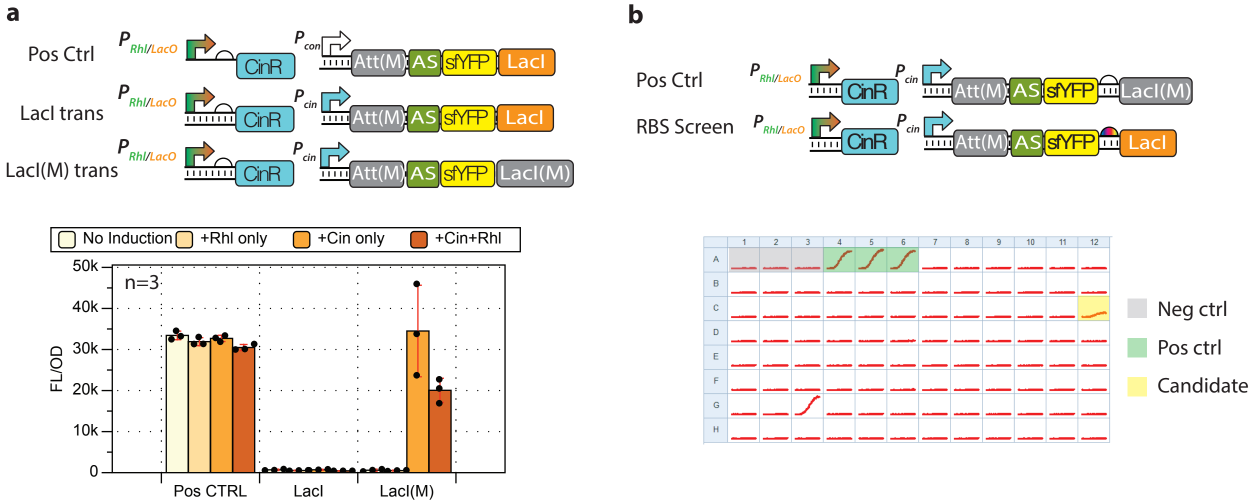

Supplementary Fig. 1: The functionality of a circuit construct is heavily dependent on RBS optimization. (a) The *trans* feedback activation test in the activating cascade without RBS optimization for CinR and LacI. The RBS strength for the CinR gene appears to be excessive as the leaky expression of CinR mRNA allows activation of the sfYFP cassette with AHL-Cin only induction (LacI(M), +Cin only). The RBS strength for the LacI gene also appears to be excessive as the cells with LacI *trans* design shows no signal across all four induction cases. The experiment also confirms that LacI(M) is a proper control for the LacI regulator. Data in this figure are presented as mean value +/- standard deviation of n samples, n = number of biological replicates. (b) The RBS screening setup for the search for a proper RBS for LacI. Each 96 well-plate was seeded with three wells of negative control cells, which contained empty antibiotic-resistant plasmids, and three wells of positive control cells, which were transformed with the open-loop construct. Ninety colonies were picked from an agar plate to inoculate 90 individual cultures. All cultures were induced with AHL-Cin and Rhl-AHL. An ideal RBS should facilitate a circuit dynamic with a signal lower than the positive control but higher than the negative controls, as highlighted in yellow.

2.2 Growth profile of the synthetic network-hosting cells

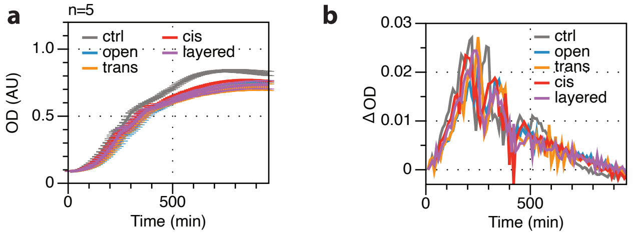

Supplementary Fig. 2: Growth rate is not constant in the circuit dynamic window. (a) The OD dynamics for experiment shown in Figure 4C. Data in this plot are presented as mean values +/- standard deviations of n samples, n = number of biological replicates. (b) The rate of growth over time for experiment in Figure 4C. Single sample traces.

3 Supplementary Simulation Results

3.1 Impulse response analysis using a linearized state space model

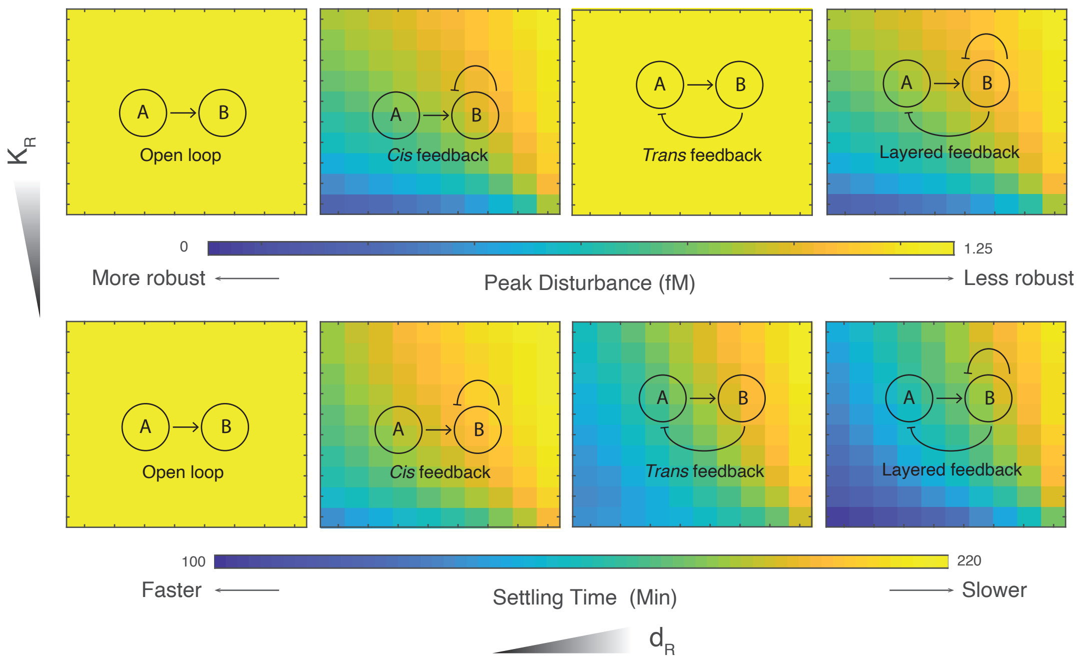

Supplementary Fig. 3: Peak disturbance and settling time for an impulse disturbance in a two-dimensional parameter space defined by K_R and d_R. The top panel shows the output peak disturbance when all three species' production rates are subjected to an impulse perturbation. The bottom panel shows the time it takes for the systems to settle back to equilibrium after the perturbation. The color schemes represent both response magnitude and settling time (see color bar). For each of the eight heat maps, the x-axis (d_R) represents the degradation rate of regulator R; a large d_R models a regulator with a fast degradation rate. The y-axis (K_R) represents the repression constant of regulator R; a large K_R models a regulator with weak repression strength.

3.2 Simulated dynamics using generic biomolecular model with parameter randomization

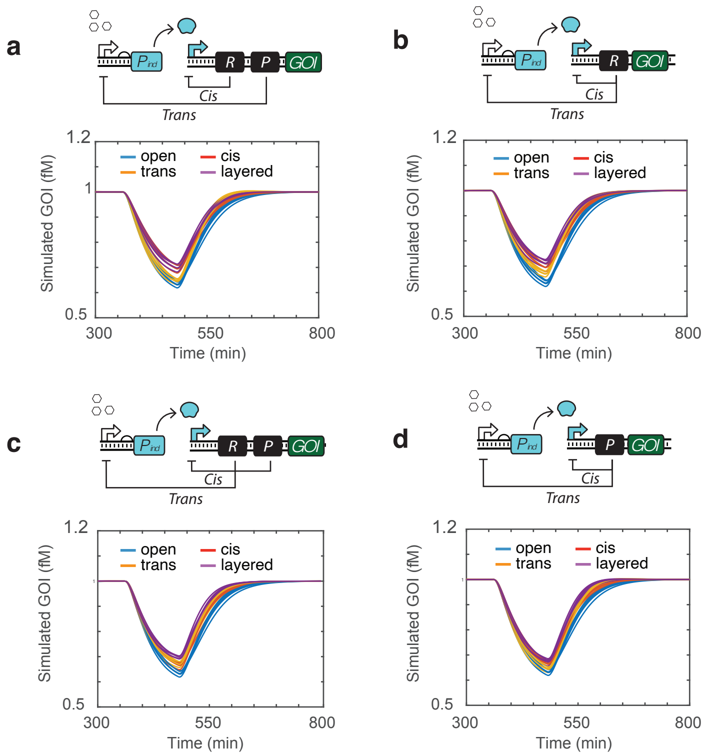

Supplementary Fig. 4: The simulated dynamics of the system with a 25% transcriptional impulse drop for 120 minutes at the system's equilibrium. All parameters are subjected to a 25% randomization. Each trajectory is scaled by its corresponding equilibrium. The performance profile in the speed-robustness domain of each design is plotted in Figure 2b.

4 Dynamical Perturbation Experiments

4.1 Data exclusion procedures based on growth profile

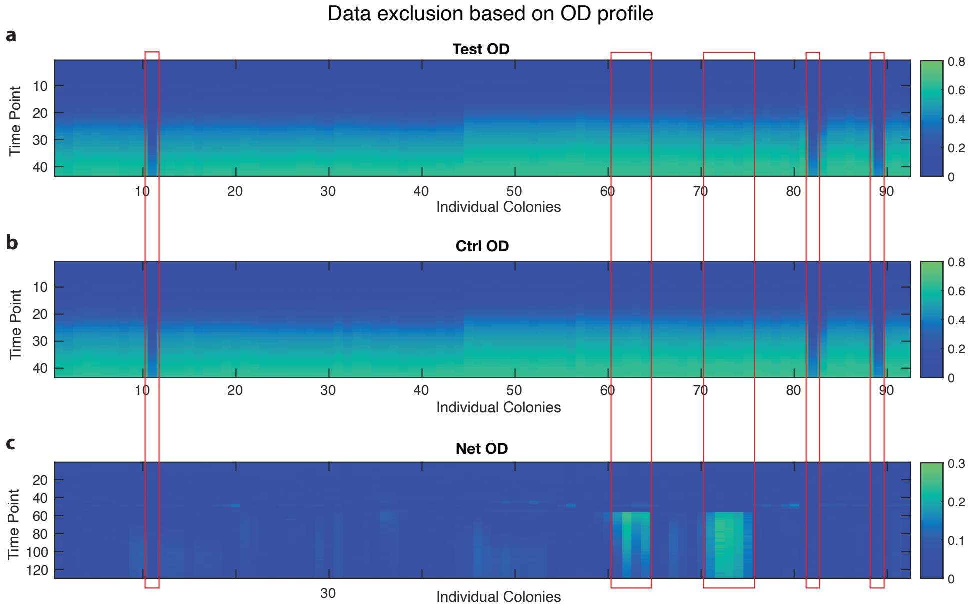

Supplementary Fig. 5: Data exclusion based on the growth profiles of individual cultures. The figure shows the optical density (OD) profiles of all 92 cultures measured in the experiment shown in Figure 5. There were 20 replicates for the open-loop, 24 replicates for each of the *trans* feedback, *cis* feedback, and the layered feedback constructs. (a) The top panel shows the OD profile of the test plate before disturbance; (b) the middle panel shows the OD profile of the control plate (intact group) before the chemical perturbation; (c) the bottom panel shows the OD difference of each culture between the perturbed and intact plates. If a culture has an outstanding growth profile in (a) and (b) or an outstanding OD difference in (c), the entire column of data is then excluded in data analysis, as marked in red boxes.

4.2 System dynamics under various types of perturbations

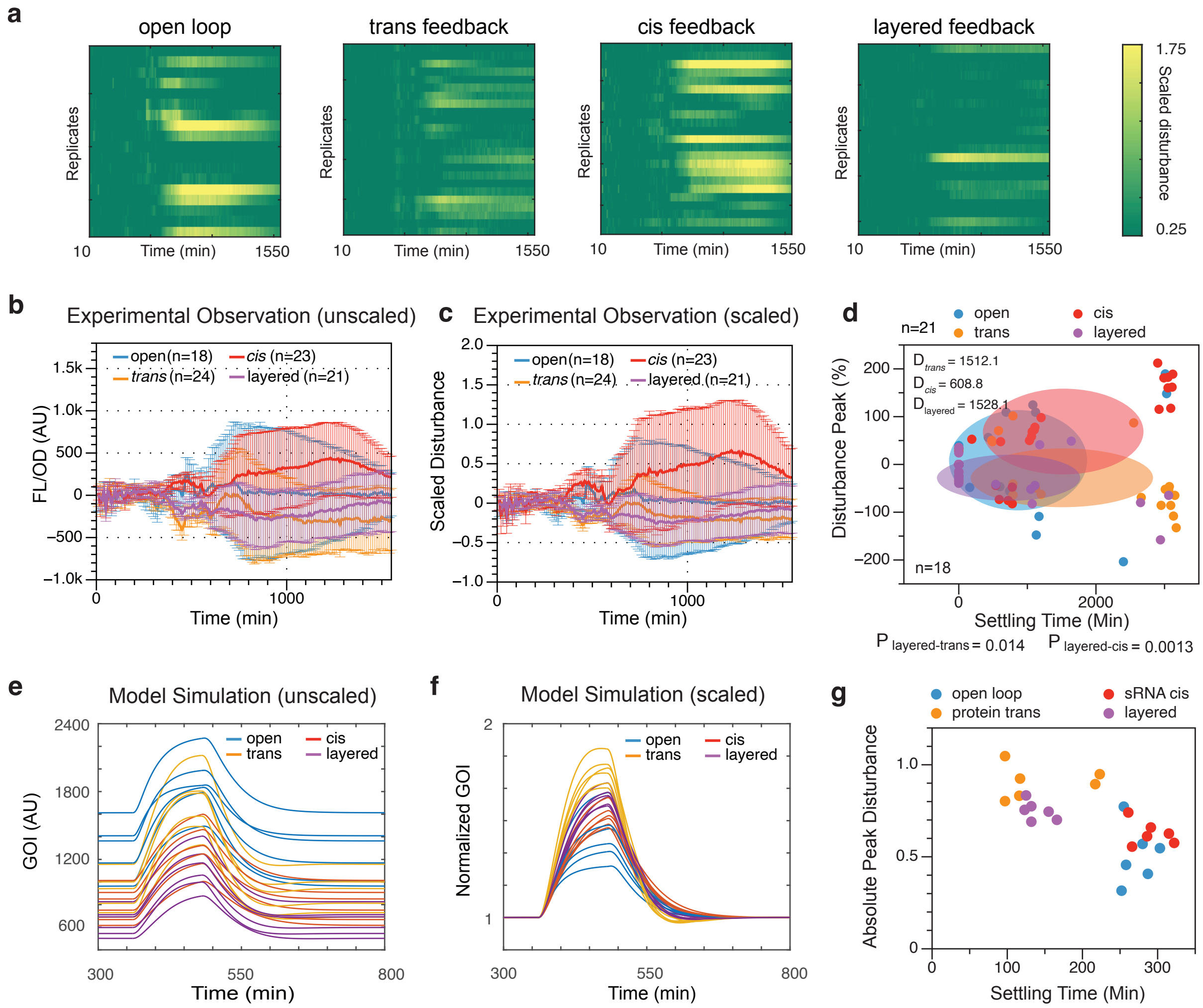

Supplementary Fig. 6: Disturbance profile with both AHL-Cin and AHL-Rhl spike. (a) The dynamical profile of four network architectures with all replicates. Each row in an individual heat map represents the scaled disturbance dynamics measured from a single liquid culture well. Each column represents the measurements at a certain time point. The color intensity indicates the absolute magnitude of disturbance output. The four heat maps illustrate the time domain disturbance profiles of the open loop, the *trans* feedback, the *cis* feedback, and the layered feedback constructs when all systems are subjected to an inducer chemical concentration perturbation. Here the inducers AHL-Cin increased from 3μM to 10 μM; the AHL-Rhl increased from 20 μM to 100 μM at 310 min and restored to the pre-disturbance media at 470 min. (b) Disturbance profile (the dynamical deviation of the perturbed group from the intact group), (c) The scaled disturbance profile (calculated with the disturbance profile scaled by each well's pre-disturbance output values). Data in (b) and (c) are presented as mean value +/- standard deviation of n samples, n = number of biological replicates. (d) The robustness-speed trade-off of the four constructs. The layered control architecture shows the most optimal performance in robustness and speed compared to the two single-layer controllers. The distance of each point to the origin quantifies the robustness-speed performance of an individual test culture. The D-values denote the average performance of its corresponding group. The value $P_{layered-trans}$ denotes the p-value between the layered control and the *trans* control, and $P_{layered-cis}$ denotes the p-value between the layered control and the *cis* control. p-values are determined by the paired one-tail Student' t-test with n samples. n = number of biologically independent replicates. Figure (e), (f), and a (g) are simulated results using the model described in SI Section 1.3, with the translation rate linked to the total concentration of mRNA in the system. The transcriptional impulse was set to a 100 % increase for 120 minutes. Six sets of parameters generated with 25% randomization were used to emulate the biological uncertainty. (e) Simulated dynamics without scaling, showing open loop signal having higher equilibrium and peak disturbances than the systems with feedback controls. (f) Simulated dynamics with scaling. All trajectories are scaled by their equilibrium values. Due to translational capping, the scaled open loop has the lowest disturbance peak. (g) Simulated dynamics of four designs evaluated in the robustness-speed domain. In this case, the two single-layer controls do not appear to improve performance. However, the layered control architecture still overcomes the performance trade-off demonstrated by the single layers and achieves optimal performance.

4.3 Detailed perturbation experiment protocols

The six sets of perturbation experiments were performed with the protocol listed in Supplementary Table 7. For each set of experiments, an intact (Ctrl) group and a perturbed (Test) group were used. In order to isolate the impact of a defined perturbation, both groups undergo the same perturbations that are associated with experimental procedures, i.e., PBS wash, temperature change associated with perturbation introduction, or replenishment of nutrients due to the use of fresh media.

Supplementary Table 7: Perturbation experiment protocols

Experiment	Int. cond.	Disturb at	Disturbance condition	Recover at	Recovery cond.
Rhl+Cin dip Test	standard*	440 min	wash, standard -Rhl -Cin	560 min	pre-disturbance media
Rhl+Cin dip Ctrl	standard	440 min	wash, standard fresh	560 min	pre-disturbance media
Rhl+Cin spike Test	low ind.**	310 min	wash, standard fresh	470 min	wash, fresh low ind.
Rhl+Cin spike Ctrl	low ind.	310 min	wash, low ind. fresh	470 min	wash, fresh low ind.
Glucose dip Test	standard	370 min	wash, standard -glucose	500 min	pre-disturbance media
Glucose dip Ctrl	standard	370 min	wash, standard	500 min	pre-disturbance media
Glucose spike Test	0.1% glucose	370 min	continuous, 1% glucose	500 min	pre-disturbance media
Glucose spike Ctrl	0.1% glucose	370 min	continuous	500 min	pre-disturbance media
Temp dip Test	standard	380 min	continuous at 30°C	510 min	continuous standard
Temp dip Ctrl	standard	380 min	continuous	510 min	continuous standard
Temp spike Test	standard	380 min	continuous at 42°C	510 min	continuous standard
Temp spike Ctrl	standard	380 min	continuous	510 min	continuous standard

** $20\mu M$ Rhl and $3\mu M$ Cin

* M9 medium with 1% glucose, induced with $100\mu M$ Rhl and $10\mu M$ Cin growing at 37°C

4.4 Supplementary Statistical Results

Supplementary Table 8: Supplementary Statistical Results

p-values (one-tail)	Figure	Sample Size	open-Layered	trans-Layered	cis-layered
TX-drop-speed	5c	17	1.86E-05	0.007195911	0.000321488
TX-drop-robustness	5d	17	3.00E-10	0.340564872	5.30E-10
TX-spike-speed	6a	18	0.33816	0.023196	9.76E-03
TX-spike-robustness	6a	18	0.33198	2.24E-01	5.56E-04
Temp-drop-speed	6b	19	0.0092304	0.00016907	3.97E-07
Temp-drop-robustness	6b	19	0.0044297	4.45E-08	0.00011369
Temp-spike-speed	6c	19	0.00024563	0.0033215	2.00E-06
Temp-spike-robustness	6c	19	7.17E-08	1.42E-14	1.22E-09
Glucose-drop-robustness	6e	20	0.01151	7.82E-03	9.18E-02
Glucose-spike-speed	6d	20	6.93E-19	0.072102	3.99E-17
Glucose-spike-robustness	6d	20	2.57E-08	0.68366	1.29E-08

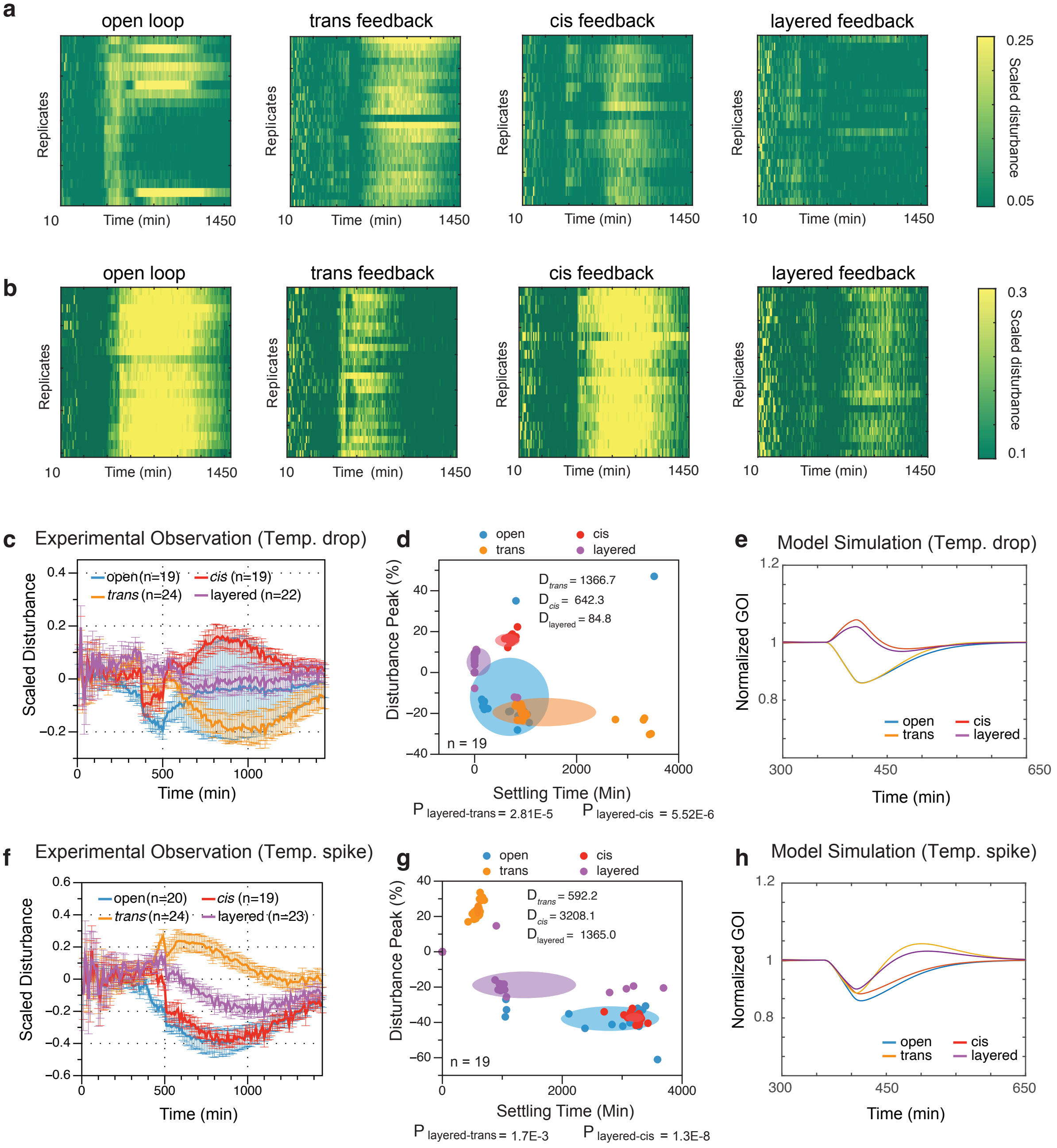

Supplementary Fig. 7: Disturbance profile with temperature perturbation in two directions. (a) The dynamical profile of the four network architectures with all included replicates when the temperature drops from 37°C to 30°C for 2 hours. (b) The dynamical profile of the four network architectures with all included replicates when temperature spikes from 37°C to 42°C for 2 hours. In each heat map, each row represents the scaled disturbance dynamics of an individual liquid culture well. Each column represents the measurements at a given time point. The color intensity indicates the absolute magnitude of disturbance output. (c) The scaled disturbance profile for temperature drop perturbation. Data in this figure are presented as mean value +/- standard deviation of n samples, n = number of biological replicates. (d) The robustness-speed performance of the four constructs with temperature drop perturbation. (e) Simulated dynamics for temperature drop using the model with sRNA regulator folding delay, as shown in section 1.2. In the model, the perturbation causes universal transcriptional slowdown, further sRNA folding delay, and the weakening of sRNA binding with increased K_R. (f) The scaled disturbance profile for temperature spike perturbation. Data in this figure are presented as mean value +/- standard deviation of n samples, n = number of biological replicates. (g) The robustness-speed performance of the four constructs with temperature spike perturbation. (h) Simulated dynamics for temperature spike using the model with sRNA regulator folding delay, as shown in section 1.2. The perturbation causes a universal transcriptional slowdown in the model, fast protein degradation, and the weakening of regulatory protein binding with increased K_p. In (d) and (g), The distance of each point to the origin quantifies the robustness-speed performance of an individual test culture. The D-values denote the average performance of its corresponding group. The value $P_{layered-trans}$ denotes the p-value between the layered control and the *trans* control, and $P_{layered-cis}$ denotes the p-value between the layered control and the *cis* control. p-values are determined by the paired one-tail Student' t-test with n samples. n = number of biologically independent replicates.

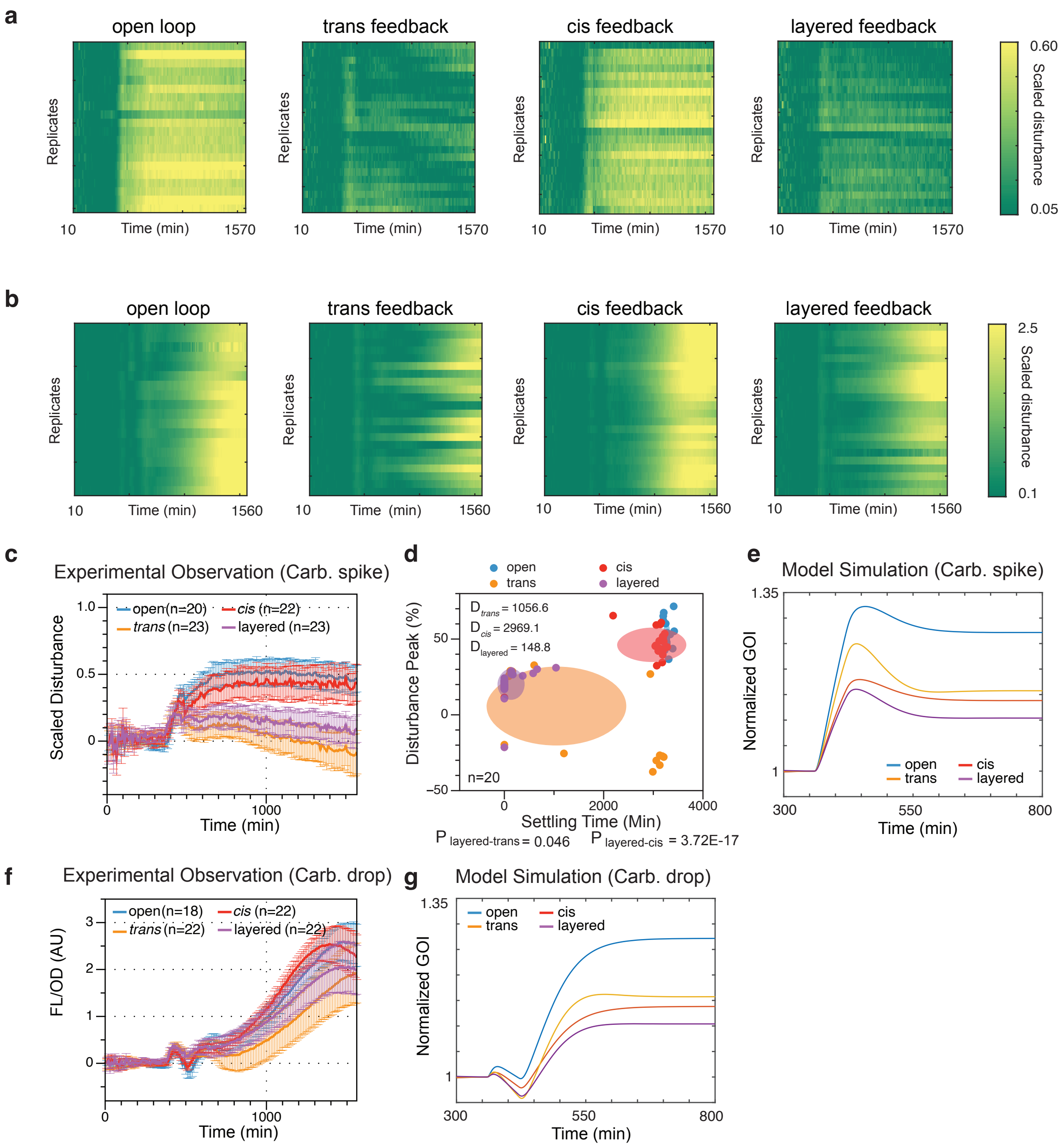

Supplementary Fig. 8: Disturbance profile with carbon source perturbation in two directions. (a) The dynamical profile of the four network architectures with all included replicates when glucose concentration increases from 0.1% to 1% for two hours. (b) The dynamical profile of the four network architectures with all included replicates when glucose concentration drops from 1% to 0% for two hours. In each heat map, each row represents the scaled disturbance dynamics of an individual liquid culture well. Each column represents the measurements at a given time point. The color intensity indicates the absolute magnitude of disturbance output. (c) The scaled disturbance profile for glucose spike perturbation presented as mean value +/- standard deviation of n samples, n = number of biological replicates. (d) The robustness-speed performance of the four constructs with glucose spike perturbation. In this figure, The distance of each point to the origin quantifies the robustness-speed performance of an individual test culture. The D-values denote the average performance of its corresponding group. The value $P_{layered-trans}$ denotes the p-value between the layered control and the *trans* control, and $P_{layered-cis}$ denotes the p-value between the layered control and the *cis* control. p-values are determined by the paired one-tail Student' t-test with n samples. n = number of biologically independent replicates. (e) Simulated dynamics for temperature drop using the model with translational capping, as shown in section 1.3. In the model, the perturbation causes a slight temporary universal transcriptional speedup and a step increase in R_{max} that does not recover, as listed in Supplementary Table 5. (f) The scaled disturbance profile for glucose drop perturbation presented as mean value +/- standard deviation of n samples, n = number of biological replicates. (g) Simulated dynamics for glucose drop using the model with translational capping, as shown in section 1.3. In the model, the perturbation causes a slight temporary universal transcriptional slowdown and a step increase in R_{max} that does not recover, as listed in Supplementary Table 5.

5 Parameter estimates

Transcription in *E. coli* is estimated to be 45nt/sec [1]. Assuming a typical mRNA in our network is 1000 nucleotide (1kb), then the transcription rate is about 2.7 counts of mRNA/min. Based on ThermoFishser's molecular conversion page [2], a single stranded 1kb RNA is about 3200g/M, and 1 μg equivalent contains about 1.88×10^{12} counts, which converts to 0.53×10^{-18} g/count. Also, we know that the volume of a single *E. coli* cell is about 1.1 μm^3 [3].

$$N_{mRNA} = \frac{1M}{3200g} \times 0.53 \times 10^{-18} g/count \approx 0.166 \times 10^{-21} M/count$$

Therefore, the transcription rate of a 1kb single stranded mRNA in *E. coli* cells can be approximated to:

$$K_{tx} = 2.7 \times (\frac{N_{mRNA}}{V_{cell}})/min = 2.7 \times (\frac{0.166 \times 10^{-21} M}{1.1 \times 10^{-6} m^3})/min \approx 0.4 \times 10^{-15} M/min = 0.4 fM/min$$

This approximates the rate of a 1kb gene transcribed from a single plasmid copy with a consensus promoter. Depending on the copy number of the plasmid of choice for carrying the genetic construct, as well as the promoter sequence in the construct, the transcription rate can differ by roughly two orders of magnitude. In Table 2, we chose the maximum transcription rates to be 2 fM/min and 20 fM/min, which are consistent with using low and medium copy plasmids and strong inducible promoters.

The translational initiation rate in *E. coli* at 37 °C is averaged at 62s [4], and the elongation rate in *E. coli* at 37 °C is estimated to be about 4-14 amino acids/s [5]. Assume a protein species in the network is about 340 AA long. On average, with minimal burden, it takes about 25 + 62 to 85 + 62 seconds to translate a protein from an mRNA template, which is about 0.4/min to 0.8/min. Depending on the ribosome binding site (RBS) sequence and the amount of mRNA in the system, this value is more likely to be smaller in synthetic biomolecular networks. Table 2 estimated the translation rate to be 0.1/min, which is realistic considering a medium-strength RBS and small to medium copy plasmids.

The mRNA degradation rate ranges from 5-10 minutes [6]. In Table 2, we estimated the degradation rate of mRNA to be 0.1/min to take account of the possible mRNA crowding caused by plasmid-encoded gene expression. Small RNAs are significantly shorter, and they degrade faster than mRNA [7]. We estimated the degradation rate of the sRNA to be 0.3/min.

The protein degradation rate in actively dividing cells is dominated by cell division. The estimated protein degradation rate of 0.03/min is realistic with a 30-minute doubling time if we consider the cells' doubling time to be 20 to 40 minutes.

The protein maturation rate was estimated to be 0.2/min based on the super-folder green fluorescent protein, which was estimated to mature within 6 minutes [8] in *E. coli.*

References

[1] J. Yu, J. Xiao, X. Ren, K. Lao, and X. S. Xie, "Probing Gene Expression in Live Cells, One Protein Molecule at a Time," *Science*, vol. 311, no. 5767, pp. 1600–1603, 2006.

[2] "DNA and RNA Molecular Weights and Conversions." [Online]. Available: https://www.thermofisher.com/us/en/home/references/ambion-tech-support/rna-tools-and-calculators/dna-and-rna-molecular-weights-and-conversions.html

[3] H. Yamada, M. Yamaguchi, K. Shimizu, S. Y. Murayama, S. Mitarai, C. Sasakawa, and H. Chibana, "Structome analysis of Escherichia coli cells by serial ultrathin sectioning reveals the precise cell profiles and the ribosome density," *Microscopy*, vol. 66, no. 4, pp. 283–294, 2017.

[4] M. Siwiak and P. Zielenkiewicz, "Transimulation - Protein Biosynthesis Web Service," *PLoS ONE*, vol. 8, no. 9, p. e73943, 2013.

[5] S. Proshkin, A. R. Rahmouni, A. Mironov, and E. Nudler, "Cooperation Between Translating Ribosomes and RNA Polymerase in Transcription Elongation," *Science*, vol. 328, no. 5977, pp. 504–508, 2010.

[6] Y. Taniguchi, P. J. Choi, G.-W. Li, H. Chen, M. Babu, J. Hearn, A. Emili, and X. S. Xie, "Quantifying E. coli Proteome and Transcriptome with Single-Molecule Sensitivity in Single Cells," *Science*, vol. 329, no. 5991, pp. 533–538, 2010.

[7] C. Y. Hu, J. D. Varner, and J. B. Lucks, "Generating effective models and parameters for rna genetic circuits," *ACS synthetic biology*, vol. 4, no. 8, pp. 914–926, 2015.

[8] J. A. Megerle, G. Fritz, U. Gerland, K. Jung, and J. O. Rädler, "Timing and Dynamics of Single Cell Gene Expression in the Arabinose Utilization System," *Biophysical Journal*, vol. 95, no. 4, pp. 2103–2115, 2008.
