# Supplementary material for: Layered feedback control overcomes performance trade-off in synthetic biomolecular networks
Source: Nat Commun. 2022 Sep 14;13:5393. doi: 10.1038/s41467-022-33058-6 (PMC9474519; doi:10.1038/s41467-022-33058-6)
Supplement: Supplementary file 2 — Reporting Summary [file 41467_2022_33058_MOESM2_ESM.pdf]

nature portfolio

Corresponding author(s): Chelsea Y. Hu

Last updated by author(s): 8/21/2022

Reporting Summary

Nature Portfolio wishes to improve the reproducibility of the work that we publish. This form provides structure for consistency and transparency in reporting. For further information on Nature Portfolio policies, see our Editorial Policies and the Editorial Policy Checklist.

Statistics

For all statistical analyses, confirm that the following items are present in the figure legend, table legend, main text, or Methods section.

n/a	Confirmed	
☐	☒	The exact sample size (n) for each experimental group/condition, given as a discrete number and unit of measurement
☐	☒	A statement on whether measurements were taken from distinct samples or whether the same sample was measured repeatedly
☐	☒	The statistical test(s) used AND whether they are one- or two-sided<br>*Only common tests should be described solely by name; describe more complex techniques in the Methods section.*
☐	☒	A description of all covariates tested
☒	☐	A description of any assumptions or corrections, such as tests of normality and adjustment for multiple comparisons
☐	☒	A full description of the statistical parameters including central tendency (e.g. means) or other basic estimates (e.g. regression coefficient) AND variation (e.g. standard deviation) or associated estimates of uncertainty (e.g. confidence intervals)
☐	☒	For null hypothesis testing, the test statistic (e.g. F, t, r) with confidence intervals, effect sizes, degrees of freedom and P value noted<br>*Give P values as exact values whenever suitable.*
☒	☐	For Bayesian analysis, information on the choice of priors and Markov chain Monte Carlo settings
☒	☐	For hierarchical and complex designs, identification of the appropriate level for tests and full reporting of outcomes
☒	☐	Estimates of effect sizes (e.g. Cohen's d, Pearson's r), indicating how they were calculated

Our web collection on statistics for biologists contains articles on many of the points above.

Software and code

Policy information about availability of computer code

Data collection	Gen5 3.04
Data analysis	All data were analyzed with Excel or MATLAB R2019a ttest.

For manuscripts utilizing custom algorithms or software that are central to the research but not yet described in published literature, software must be made available to editors and reviewers. We strongly encourage code deposition in a community repository (e.g. GitHub). See the Nature Portfolio guidelines for submitting code & software for further information.

Data

Policy information about availability of data

All manuscripts must include a data availability statement. This statement should provide the following information, where applicable:

- Accession codes, unique identifiers, or web links for publicly available datasets
- A description of any restrictions on data availability
- For clinical datasets or third party data, please ensure that the statement adheres to our policy

All source data and source code generated with this study is provided in the supplementary file.

Field-specific reporting

Life sciences study design

All studies must disclose on these points even when the disclosure is negative.

Sample size	Three to six biological replicates were included for circuit part-characterization (Figure 3, 4 and Figure S1, S2). No sample size calculation was performed. The replicates showed high reproducibilities to confirm circuit functions by parts.<br>Six different perturbation methods were used to experimentally confirm the robustness-speed advantageous performance of layered control (Figure 5 and Figure 6). Each set of experiment includes 20-24 biological replicates. No sample size calculation was performed, the sample size was limited by the instrument capacity. The authors maximized the sample size under instrumental current limitation.
Data exclusions	In Figure 5 and 6, data traces with atypical growth profile or outstanding OD difference between the perturbation plate and the control plate are excluded (See Figure S2).<br>-Figure 5 experiment: 2/20 traces from the open loop group, 4/24 traces from the cis feedback group, and 7/24 traces from the layered feedback group were excluded, due to atypical growth profile.<br>-Figure 6A: 2/20 traces from the open loop group, 1/24 traces from the cis feedback group, and 3/24 traces from the layered feedback group were excluded, due to atypical growth profile.<br>-Figure 6B: 1/20 trace from the open loop group, 5/24 tracec from the cis feedback group, and 2/24 traces from the layered feedback group were excluded, due to atypical growth profile.<br>-Figure 6C: 5/24 traces from the cis feedback group, and 1/24 trace from the layered feedback group were excluded, due to atypical growth profile.<br>-Figure 6D: 1/24 trace from the trans feedback group, 2/24 traces from the cis feedback group, and 1/24 trace from the layered feedback group were excluded, due to atypical growth profile.<br>-Figure 6E: 1/20 traces from the open loop group, 2/24 traces from the trans feedback group, 2/24 traces from the cis feedback group, and 1/24 trace from the layered feedback group were excluded, due to atypical growth profile. Additionally, 1/20 from the open loop group and 1/24 from the layered feedback group were also excluded due to outstandingly weak fluorescent signal.
Replication	Experiments were performed with 3-6 biological replicates in Figure 3-4, and between 18-24 biological replicates in Figure 5-6. Each replicate results from an individual colony. The colonies come from plated cells transformed with purified circular DNA using antibiotics as selection markers. All replicates were successfully measured and included in the source data.
Randomization	All single colonies were picked randomly from agar plates where the cells where transformed with purified circular DNA using antibiotics as selection markers. No manual allocation methods were used. Each plate resulted from a single transformation and all colonies were assumed to be biological replicates. In the disturbance experiments, we transformed 5 strains. We picked 4 colonies for blank, 20 colonies for open loop, 24 colonies for trans, cis, and layered control, respectively, and placed them in 96-well culture plates.
Blinding	Data collection was done automatically by plate readers. Data analysis was performed by software written in MATLAB R2019a. The investigators was not blinded to the preplanned locations of each group, but no manual allocation or sorting was involved during analysis.

Reporting for specific materials, systems and methods

We require information from authors about some types of materials, experimental systems and methods used in many studies. Here, indicate whether each material, system or method listed is relevant to your study. If you are not sure if a list item applies to your research, read the appropriate section before selecting a response.

Materials & experimental systems

n/a	Involved in the study	
☒	☐	Antibodies
☒	☐	Eukaryotic cell lines
☒	☐	Palaeontology and archaeology
☒	☐	Animals and other organisms
☒	☐	Human research participants
☒	☐	Clinical data
☒	☐	Dual use research of concern

Methods

n/a	Involved in the study	
☒	☐	ChIP-seq
☒	☐	Flow cytometry
☒	☐	MRI-based neuroimaging
