# Supplementary material for: Layered feedback control overcomes performance trade-off in synthetic biomolecular networks
Source: Nat Commun. 2022 Sep 14;13:5393. doi: 10.1038/s41467-022-33058-6 (PMC9474519; doi:10.1038/s41467-022-33058-6)
Supplement: Supplementary file 4 — Source Data [file 41467_2022_33058_MOESM4_ESM.zip › Source_Data_and_Source_Code_Final_Revision/README.rtf]

The software used to generate plots in each figure is in its corresponding folder. All software are written in MATLAB_R2019a.Download and install :https://www.mathworks.com/products/get-matlab.html?s_tid=gn_getmlFollow the instructionthe control system toolbox needs to be installed as well.It takes about 2 hours to install MATLAB.Each fold contains instructions to produce the corresponding figure with the code.All scripts should take less than 30 seconds to run
