# Supplementary material for: Layered feedback control overcomes performance trade-off in synthetic biomolecular networks
Source: Nat Commun. 2022 Sep 14;13:5393. doi: 10.1038/s41467-022-33058-6 (PMC9474519; doi:10.1038/s41467-022-33058-6)
Supplement: Supplementary file 4 — Source Data [file 41467_2022_33058_MOESM4_ESM.zip › Source_Data_and_Source_Code_Final_Revision/Figure_5&Supplementary_FigureS5/README.rtf]

Source data and its analysis are included in the excel file A11.02R35-AHLwash.xlsxTwo text files “test_profile_data_excl.txt” and “ctrl_profile_data_excl.text” were copied from the the excel file for automated data analysis.The Matlab file “Analysis_main”  was used to generate the heatmaps in Figure 5A. Figure 5B was generated using a single trace of raw data layered with a its smoothed trace computed by the MATLAB function smooth.The output text files “trade-off-mag.txt” and “trade-off-time.txt” were used to generate Figure 5 C, D, E.After data exclusion, there are 18 ctrl, 24 trans, 20 cis and 17 layered in the dataDisturbance ranges from 440-550.Figure S3 was generated with data in this experiment, its analysis is in its own folder
