# Supplementary material for: Layered feedback control overcomes performance trade-off in synthetic biomolecular networks
Source: Nat Commun. 2022 Sep 14;13:5393. doi: 10.1038/s41467-022-33058-6 (PMC9474519; doi:10.1038/s41467-022-33058-6)
Supplement: Supplementary file 4 — Source Data [file 41467_2022_33058_MOESM4_ESM.zip › Source_Data_and_Source_Code_Final_Revision/Figure_6&Supplementary_FigureS6-S8/README.rtf]

Source data and its analysis is included in the excel file in each folderTwo text files “test_profile_data_excl.txt” and “ctrl_profile_data_excl.text” were copied from the the excel file for automated data analysis.The Matlab file “Analysis_main”  was used to generate the heatmaps in each experiment.The output text files “trade-off-mag.txt” and “trade-off-time.txt” were used to generate Figure the trade off plots, the robustness comparison plot, and the speed comparison plot.Figure 6A and Figure S4 were generated using data and code in folder “A.11.02R36-AHLspike-24X”Figure 6B and Figure S5 were generated using data and code in folder “A.11.02R39-Temp-2directions-24X” - “30C”Figure 6C and Figure S6 were generated using data and code in folder “A.11.02R39-Temp-2directions-24X” - “42C”Figure 6D and Figure S7 were generated using data and code in folder “A.11.02R43-Glucose-spike-24X” Figure 6E and Figure S8 were generated using data and code in folder “A.11.02R42-Glucose-dip-24X” 
