# Supplementary material for: Layered feedback control overcomes performance trade-off in synthetic biomolecular networks
Source: Nat Commun. 2022 Sep 14;13:5393. doi: 10.1038/s41467-022-33058-6 (PMC9474519; doi:10.1038/s41467-022-33058-6)
Supplement: Supplementary file 4 — Source Data [file 41467_2022_33058_MOESM4_ESM.zip › Source_Data_and_Source_Code_Final_Revision/Figure_4&Supplementary_FigureS2/Figure4D/texttype.rtf]

% $M_cin$   & Cin synthase mRNA            \frac{dM_{cin}}{dt} &=& \beta_{rhl} \cdot (\frac{X_{rhl}}{X_{rhl}+K_{rhl}}+l_{rhl})* f_{trans}-d_m \cdot M_{cin}+k_r \cdot T_{cin}-M_{cin} \cdot B \cdot k_{tf}+T_{cin} \cdot k_{{cin}_R}};% $P_cin$   & Signaling protein translated peptides (cinR)    \frac{dP_{cin}}{dt} &=& k_r \cdot T_{cin}-m_r \cdot P_{cin}-d_g \cdot P_{cin}-d_p \cdot P_{cin} ;% $C_{act}$   &   folded cinR protein bound with AHL     \frac{dC_{act}}{dt}&=& m_r \cdot P_{cin}-d_g \cdot C_{act}-d_p \cdot C_{act};% $R$ Reguator sRNA     \frac{dR}{dt}&=& \beta_{cin} \cdot (\frac{C_{act}}{C_{act}+K_{cin}}+l_{cin}) \cdot f_{cis}-d_r \cdot R-m_as \cdot R;% $M$   & Regulator and sfYFP mRNA    \frac{dM}{dt}&=& \beta_{cin} \cdot (\frac{C_{act}}{C_{act}+K_{cin}}+l_{cin}) \cdot f_{cis}-d_m \cdot M+k_r \cdot T_{lac}-M \cdot B \cdot k_{tf}+T_{lac} \cdot k_{{lac}_R}}+k_r \cdot T_{fp}-M \cdot B \cdot k_{tf}+T_{fp} \cdot k_{{fp}_R}};% $P_lac$ Regulator protein lacI    \frac{dP_{lac}}{dt}&=& k_r \cdot T_{lac}-d_g \cdot P_{lac}-d_p \cdot P_{lac}-m_{lac} \cdot P_{lac};    % $B$   & ribosome     \frac{dB}}{dt} &=& -M_{cin} \cdot B \cdot k_{tf}+T_{cin} \cdot k_{{cin}_R}}-2 \cdot M \cdot B \cdot k_{tf}+T_{lac} \cdot k_{{lac}_R}}+T_{fp} \cdot k_{{fp}_R}}+k_r \cdot (T_{cin}+T_{lac}+T_{fp})-d_p \cdot B;% $G$   & translated sfYFP peptides   \frac{dG}}{dt}&=& k_r \cdot T_{fp}-alpha \cdot P_{fp}-d_g \cdot P_{fp}-d_p \cdot P_{fp};% $T_cin$ & rbs bound cin mRNA complex   \frac{dT_{cin}}{dt}&=& -k_r \cdot T_{cin}+M_{cin} \cdot B\cdot k_{tf}-T_{cin} \cdot k_{{cin}_R}}-d_g \cdot T_{cin};% $T_lac$ & rbs bound lacI mRNA complex    \frac{dT_{lac}}{dt}&=& -k_r \cdot T_{lac}+M \cdot B \cdot k_{tf}-T_{lac} \cdot k_{{lac}_R}}-d_g \cdot T_{lac};% $T_fp$ & rbs bound yfp mRNA complex    \frac{dT_{fp}}{dt}&=& -k_r \cdot T_{fp}+M \cdot B \cdot k_{tf}-T_{fp} \cdot k_{{fp}_R}}-d_g \cdot T_{fp};% $R_m$ mature sRNA       \frac{dR_m}{dt}&=& m_as \cdot R-d_r \cdot R_m;% $P_{m_{lac}}$ mature lacI     \frac{dP_{m_{lac}}}}{dt}&=& m_lac \cdot P_{lac}-d_g \cdot P_{m_{lac}}-d_p \cdot P_{m_{lac}};  % C & cell density         \frac{dC}{dt}&=& r_g \cdot (1-\frac{C}{C_{max}}) \cdot C;% $P_{fp}$   & matured sfYFP,system signal    \frac{dP_{fp}}{dt} &=& alpha \cdot G-d_g \cdot P_{m_{fp}}-d_p \cdot P_{fp};
