# Supplementary material for: Layered feedback control overcomes performance trade-off in synthetic biomolecular networks
Source: Nat Commun. 2022 Sep 14;13:5393. doi: 10.1038/s41467-022-33058-6 (PMC9474519; doi:10.1038/s41467-022-33058-6)
Supplement: Supplementary file 4 — Source Data [file 41467_2022_33058_MOESM4_ESM.zip › Source_Data_and_Source_Code_Final_Revision/Figure_2&Supplementary_FigureS4/Figure 2A/README.rtf]

In each folder, open “Sim_disturbance_window_Main.m”For the sRNA-sRNA and Protein-protein mediated designs, each parameter scan cover 5X5 parameter space.For the RcisPtrans and RtransPcis designs, the parameter scan cover 5X5X2 parameter space. open “Datafile_perturbation_P_space.m”. In the parameter space scan section, there are two sets of code for scanning. Comment out protein scan for sRNA, and comment out sRNA for protein.To plot both protein scan and RNA scan on the same plot:Run  “Sim_disturbance_window_Main.m”  with protein parameter scan in “Datafile_perturbation_P_space.m”. Then, comment out the first two lines in the “Sim_disturbance_window_Main.m” file and run again with RNA parameter scan in “Datafile_perturbation_P_space.m”.
