# Supplementary material for: Layered feedback control overcomes performance trade-off in synthetic biomolecular networks
Source: Nat Commun. 2022 Sep 14;13:5393. doi: 10.1038/s41467-022-33058-6 (PMC9474519; doi:10.1038/s41467-022-33058-6)
Supplement: Supplementary file 4 — Source Data [file 41467_2022_33058_MOESM4_ESM.zip › Source_Data_and_Source_Code_Final_Revision/Figure_2&Supplementary_FigureS4/Figure 2B/README.rtf]

In each folder, open “Sim_disturbance_window_Main.m”It outputs a text file with either time or peak.The four columns represent : open, cis, trans, layered  
