# Supplementary figures and images for: Layered feedback control overcomes performance trade-off in synthetic biomolecular networks
Source: Nat Commun. 2022 Sep 14;13:5393. doi: 10.1038/s41467-022-33058-6 (PMC9474519; doi:10.1038/s41467-022-33058-6)

FL/OD (AU)

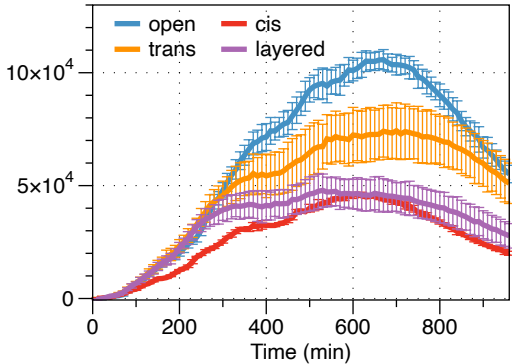

Supplement: Supplementary file 4 — Source Data [file 41467_2022_33058_MOESM4_ESM.zip › Source_Data_and_Source_Code_Final_Revision/Figure_4&Supplementary_FigureS2/Figure4B-C/A11.02R12-37.pdf]

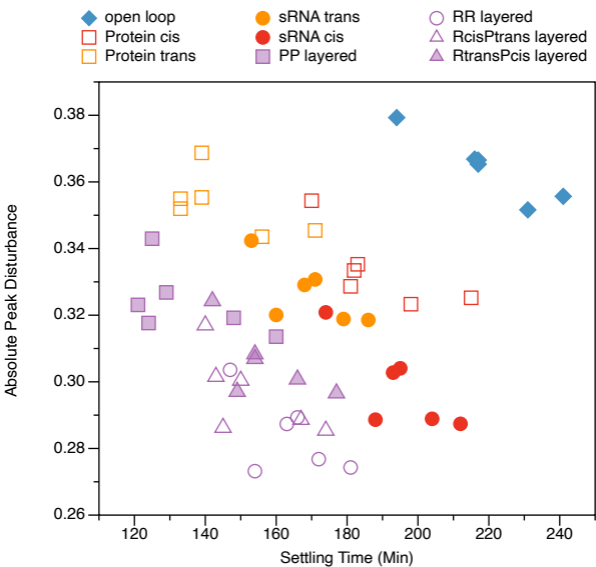

Supplement: Supplementary file 4 — Source Data [file 41467_2022_33058_MOESM4_ESM.zip › Source_Data_and_Source_Code_Final_Revision/Figure_2&Supplementary_FigureS4/Figure 2B/trade-off hybrid-tx-dip-120min.pdf]

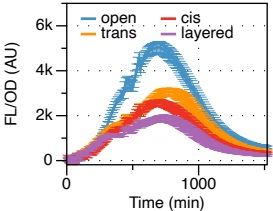

Supplement: Supplementary file 4 — Source Data [file 41467_2022_33058_MOESM4_ESM.zip › Source_Data_and_Source_Code_Final_Revision/Figure_5&Supplementary_FigureS5/Figture 5/R35ctrl.pdf]

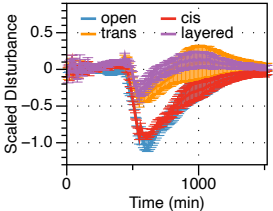

Supplement: Supplementary file 4 — Source Data [file 41467_2022_33058_MOESM4_ESM.zip › Source_Data_and_Source_Code_Final_Revision/Figure_5&Supplementary_FigureS5/Figture 5/R35-scaled_disturbance.pdf]

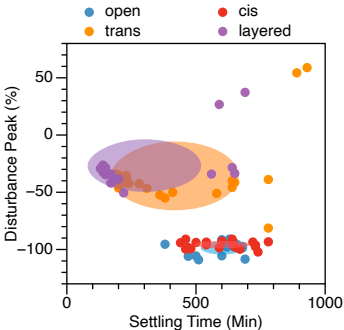

Supplement: Supplementary file 4 — Source Data [file 41467_2022_33058_MOESM4_ESM.zip › Source_Data_and_Source_Code_Final_Revision/Figure_5&Supplementary_FigureS5/Figture 5/trade-off.pdf]

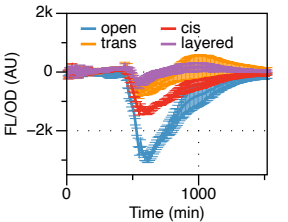

Supplement: Supplementary file 4 — Source Data [file 41467_2022_33058_MOESM4_ESM.zip › Source_Data_and_Source_Code_Final_Revision/Figure_5&Supplementary_FigureS5/Figture 5/R35-disturbance.pdf]

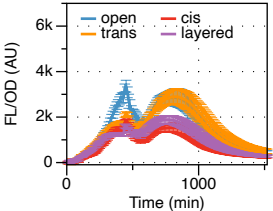

Supplement: Supplementary file 4 — Source Data [file 41467_2022_33058_MOESM4_ESM.zip › Source_Data_and_Source_Code_Final_Revision/Figure_5&Supplementary_FigureS5/Figture 5/R35test.pdf]

Settling Time (min)

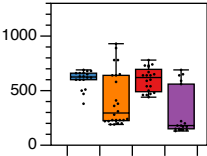

Supplement: Supplementary file 4 — Source Data [file 41467_2022_33058_MOESM4_ESM.zip › Source_Data_and_Source_Code_Final_Revision/Figure_5&Supplementary_FigureS5/Figture 5/time plots-4.pdf]

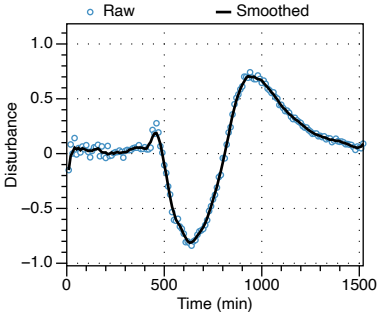

Supplement: Supplementary file 4 — Source Data [file 41467_2022_33058_MOESM4_ESM.zip › Source_Data_and_Source_Code_Final_Revision/Figure_5&Supplementary_FigureS5/Figture 5/Analysis_example.pdf]

% Disturbance peak

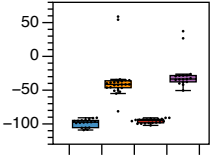

Supplement: Supplementary file 4 — Source Data [file 41467_2022_33058_MOESM4_ESM.zip › Source_Data_and_Source_Code_Final_Revision/Figure_5&Supplementary_FigureS5/Figture 5/peak plots-4.pdf]

## Data exclusion based on OD profile

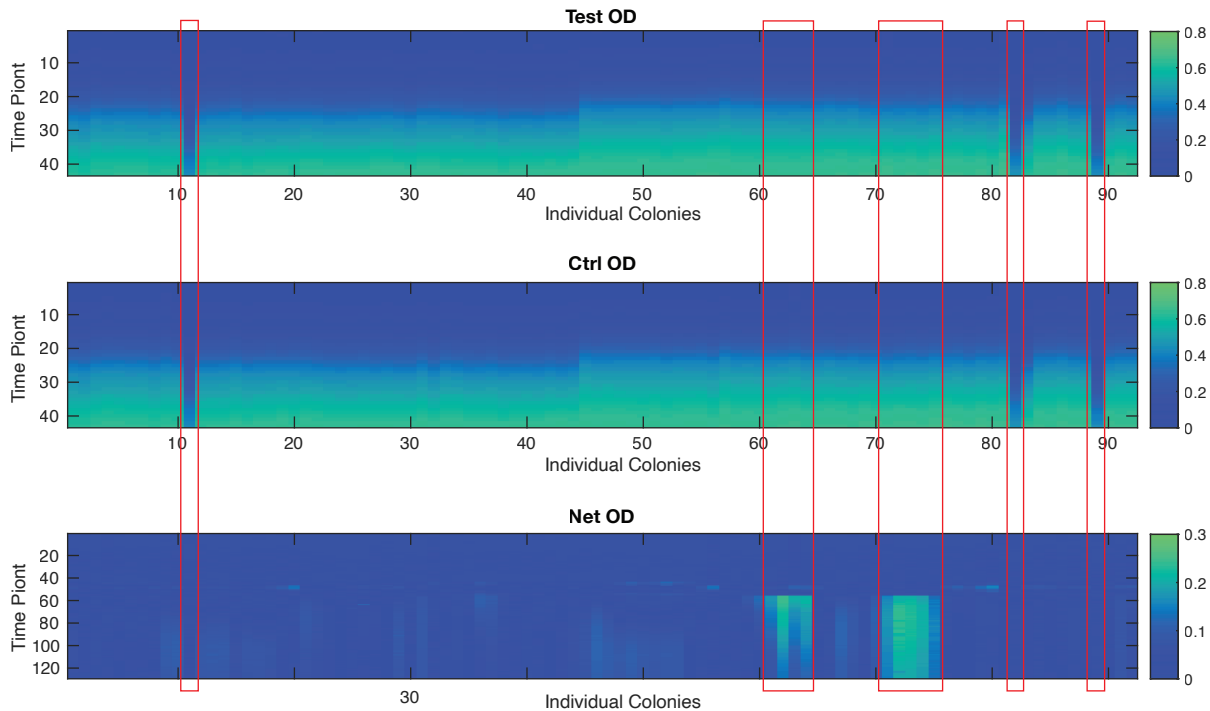

Supplement: Supplementary file 4 — Source Data [file 41467_2022_33058_MOESM4_ESM.zip › Source_Data_and_Source_Code_Final_Revision/Figure_5&Supplementary_FigureS5/Supplementary_FigureS5/SI-data-excl.pdf]

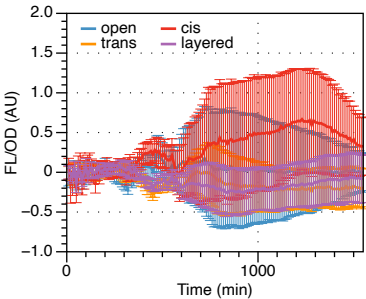

Supplement: Supplementary file 4 — Source Data [file 41467_2022_33058_MOESM4_ESM.zip › Source_Data_and_Source_Code_Final_Revision/Figure_6&Supplementary_FigureS6-S8/A11.02R36-AHLspike-24X/scaled-disturbance.pdf]

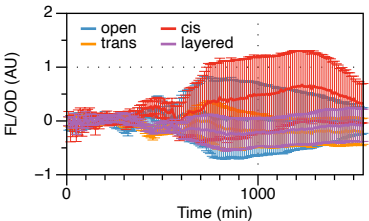

Supplement: Supplementary file 4 — Source Data [file 41467_2022_33058_MOESM4_ESM.zip › Source_Data_and_Source_Code_Final_Revision/Figure_6&Supplementary_FigureS6-S8/A11.02R36-AHLspike-24X/scaled-disturbance_2X1.2.pdf]

FL/OD (AU)

open cis  
trans layered

1.5k

1.0k

500

0

-500

-1.0k

0

1000

Time (min)

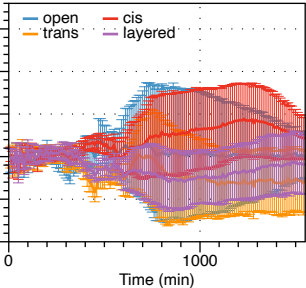

Supplement: Supplementary file 4 — Source Data [file 41467_2022_33058_MOESM4_ESM.zip › Source_Data_and_Source_Code_Final_Revision/Figure_6&Supplementary_FigureS6-S8/A11.02R36-AHLspike-24X/disturbance.pdf]

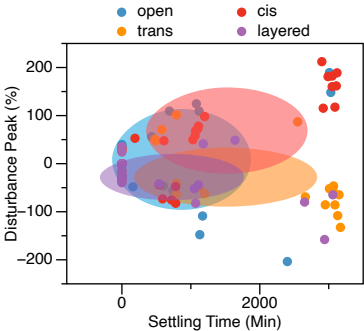

Supplement: Supplementary file 4 — Source Data [file 41467_2022_33058_MOESM4_ESM.zip › Source_Data_and_Source_Code_Final_Revision/Figure_6&Supplementary_FigureS6-S8/A11.02R36-AHLspike-24X/tradeoff.pdf]

Settling Time (min)

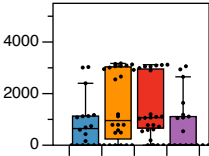

Supplement: Supplementary file 4 — Source Data [file 41467_2022_33058_MOESM4_ESM.zip › Source_Data_and_Source_Code_Final_Revision/Figure_6&Supplementary_FigureS6-S8/A11.02R36-AHLspike-24X/time plots-4.pdf]

% Disturbance peak

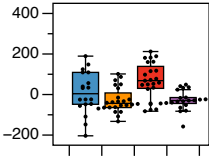

Supplement: Supplementary file 4 — Source Data [file 41467_2022_33058_MOESM4_ESM.zip › Source_Data_and_Source_Code_Final_Revision/Figure_6&Supplementary_FigureS6-S8/A11.02R36-AHLspike-24X/peak plots-4.pdf]

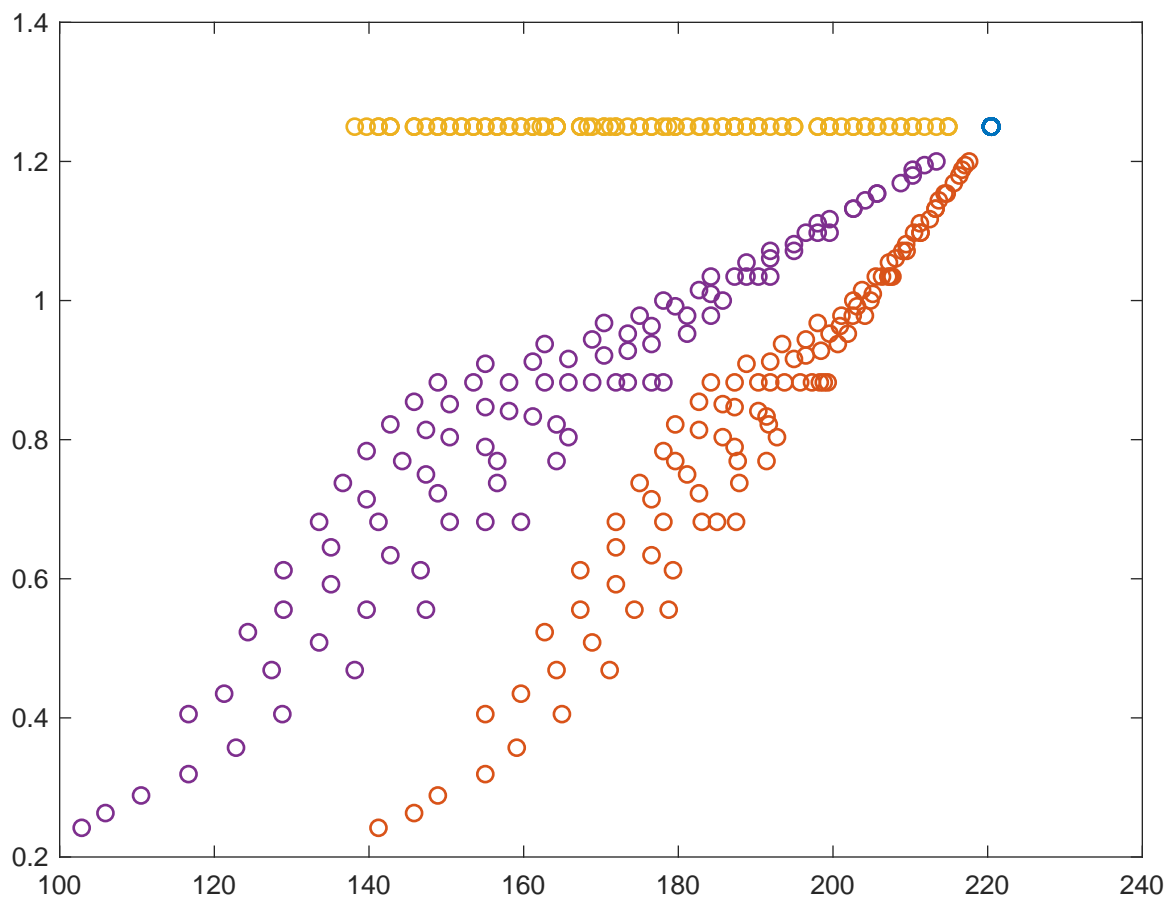

Supplement: Supplementary file 4 — Source Data [file 41467_2022_33058_MOESM4_ESM.zip › Source_Data_and_Source_Code_Final_Revision/Figure_1&Supplementary_FigureS3/Simulation-FIGURE_1/trade-off-impulse-scaled.pdf]

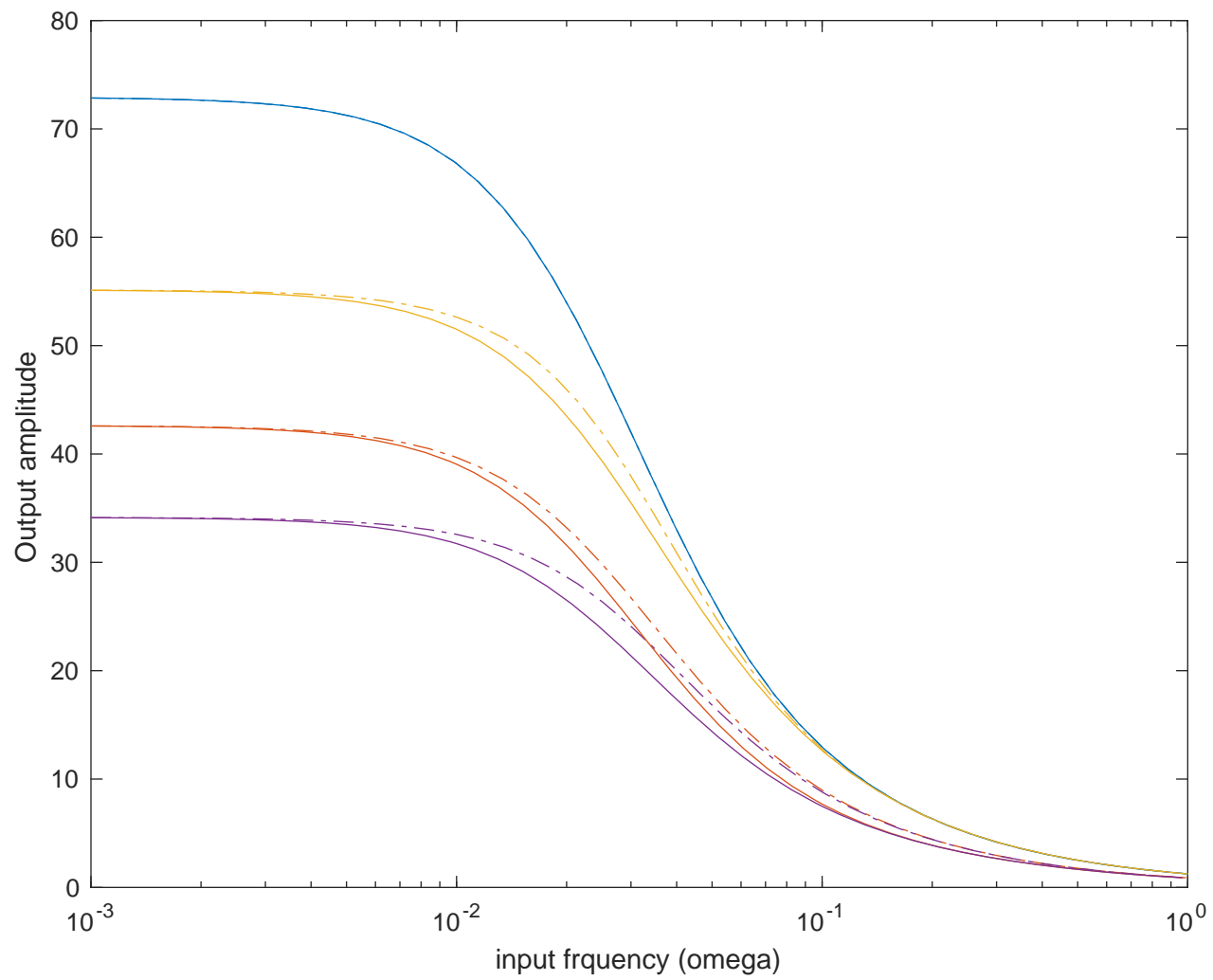

Supplement: Supplementary file 4 — Source Data [file 41467_2022_33058_MOESM4_ESM.zip › Source_Data_and_Source_Code_Final_Revision/Figure_1&Supplementary_FigureS3/Simulation-FIGURE_1/bode_mag_RNAvsProtein_log.pdf]

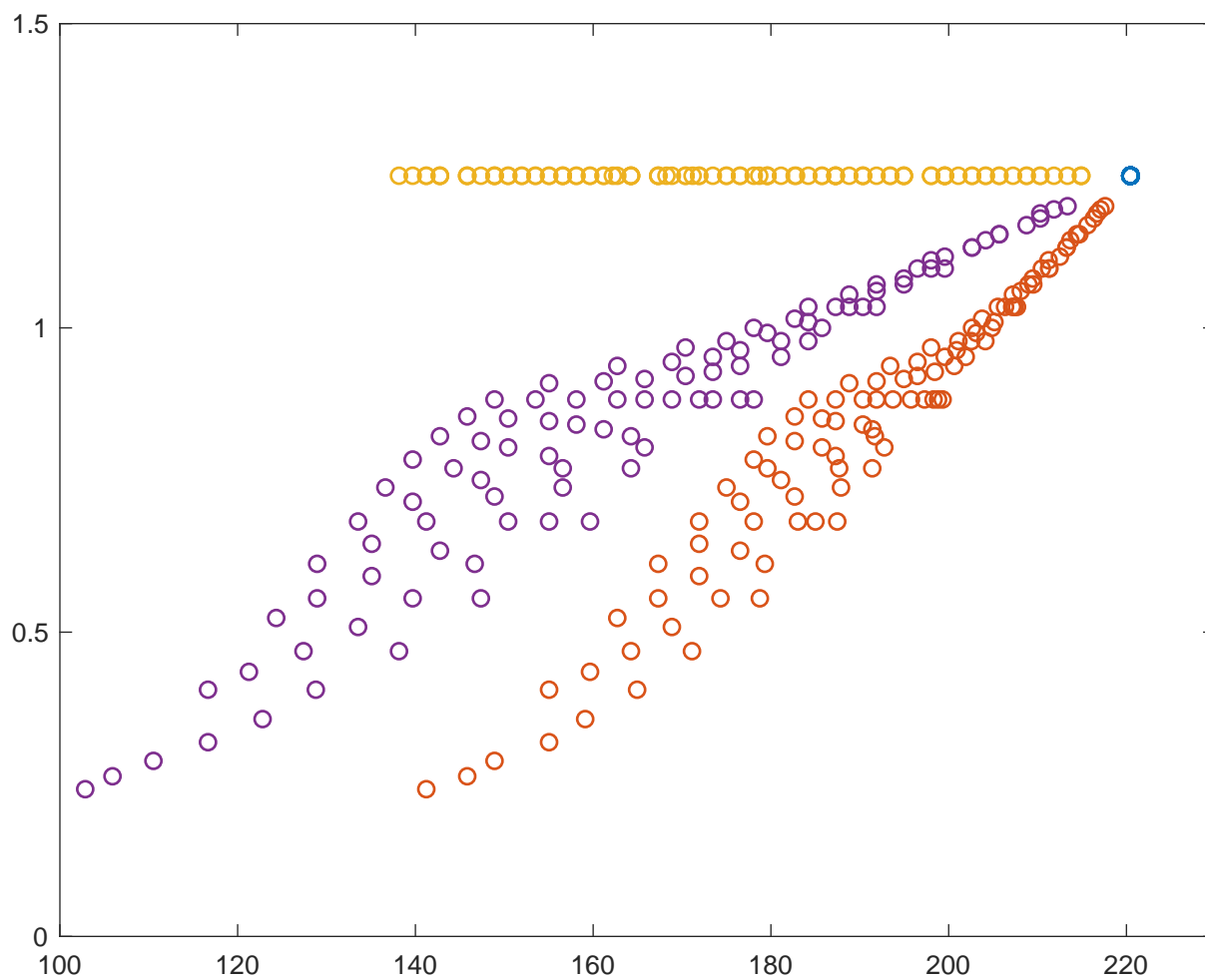

Supplement: Supplementary file 4 — Source Data [file 41467_2022_33058_MOESM4_ESM.zip › Source_Data_and_Source_Code_Final_Revision/Figure_1&Supplementary_FigureS3/Simulation-FIGURE_1/trade-off-impulse.pdf]

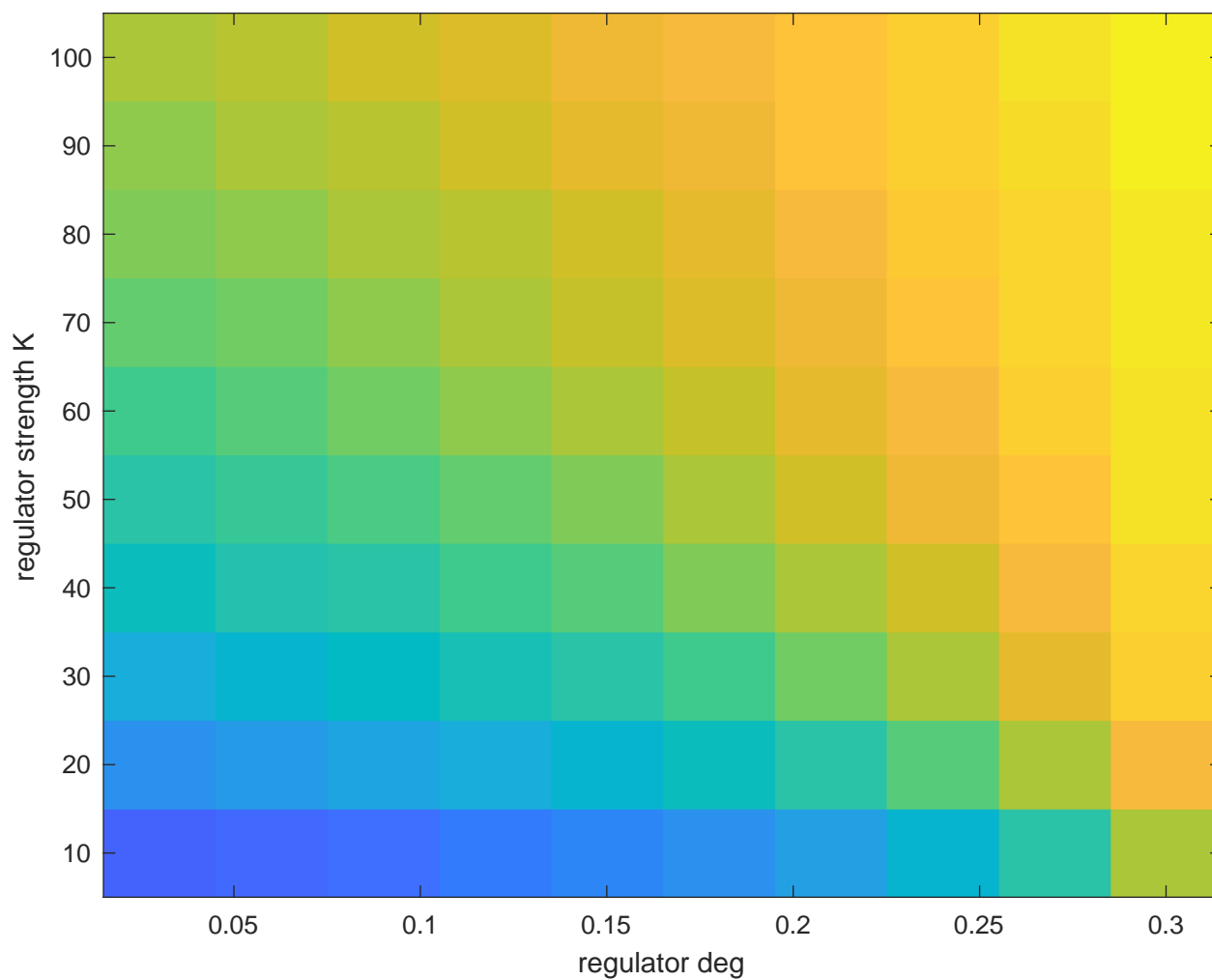

Supplement: Supplementary file 4 — Source Data [file 41467_2022_33058_MOESM4_ESM.zip › Source_Data_and_Source_Code_Final_Revision/Figure_1&Supplementary_FigureS3/Simulation-FIGURE_1/heatmap_impls_mag-4.pdf]

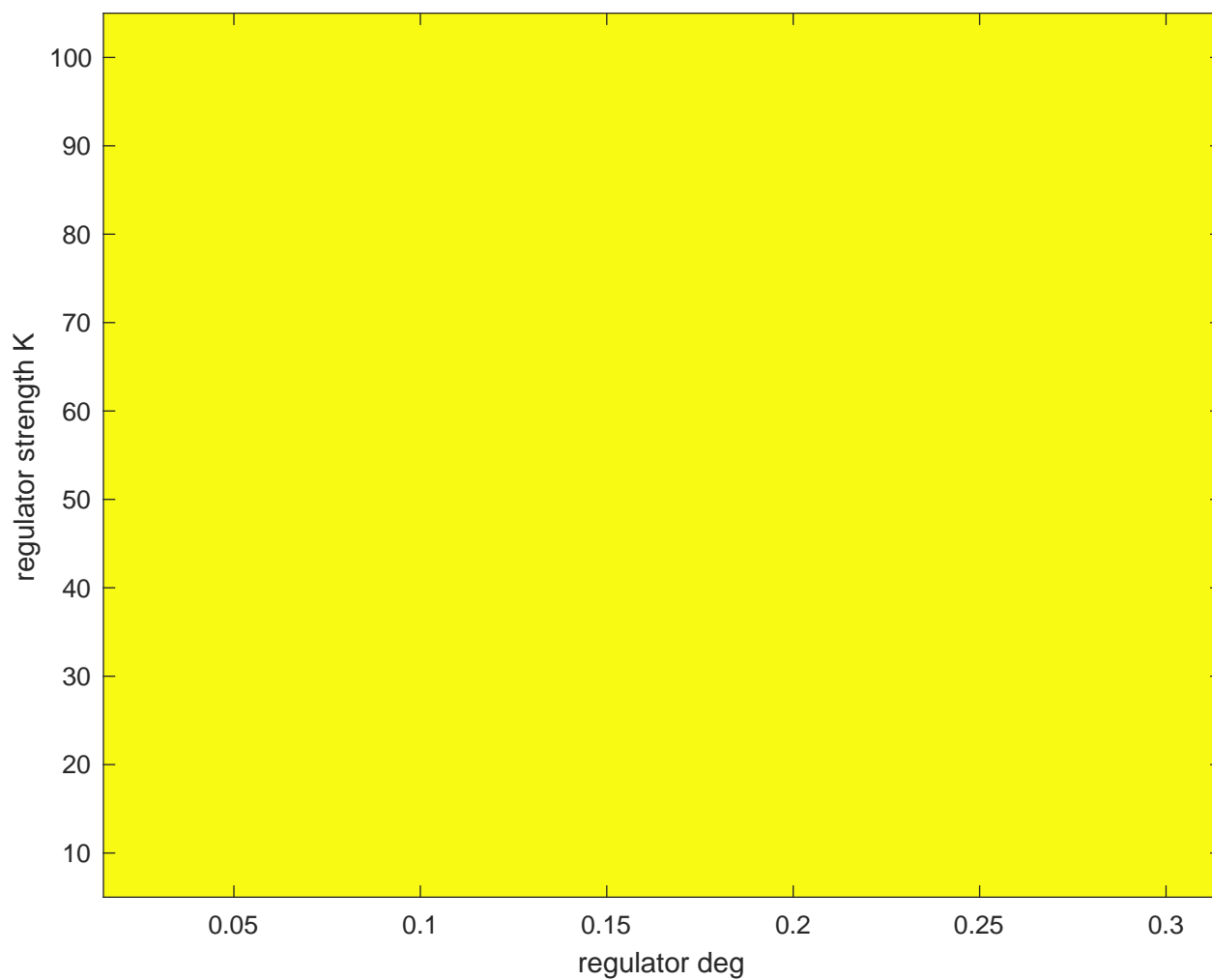

Supplement: Supplementary file 4 — Source Data [file 41467_2022_33058_MOESM4_ESM.zip › Source_Data_and_Source_Code_Final_Revision/Figure_1&Supplementary_FigureS3/Simulation-FIGURE_1/heatmap_impls_mag-3.pdf]

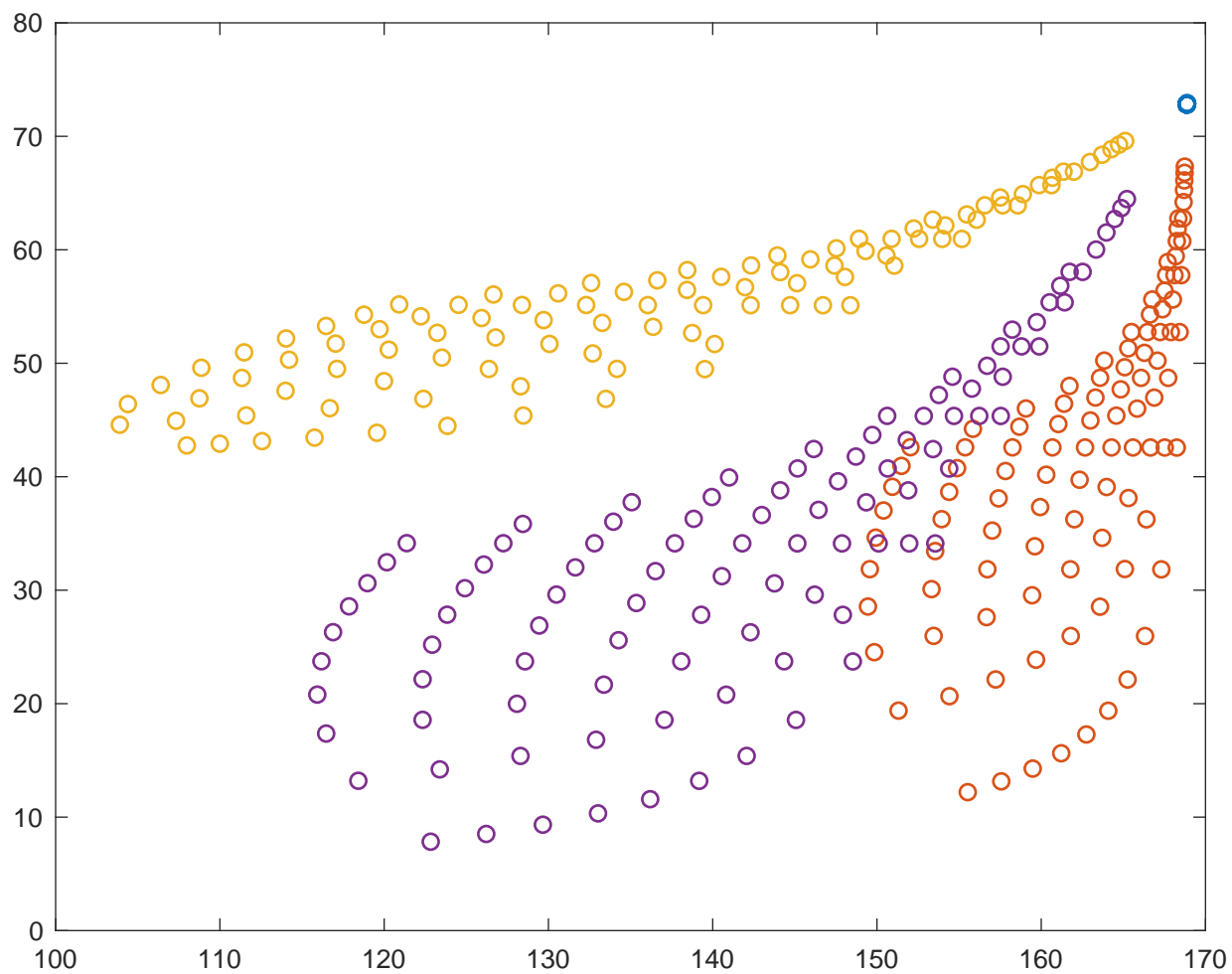

Supplement: Supplementary file 4 — Source Data [file 41467_2022_33058_MOESM4_ESM.zip › Source_Data_and_Source_Code_Final_Revision/Figure_1&Supplementary_FigureS3/Simulation-FIGURE_1/trade-off-step.pdf]

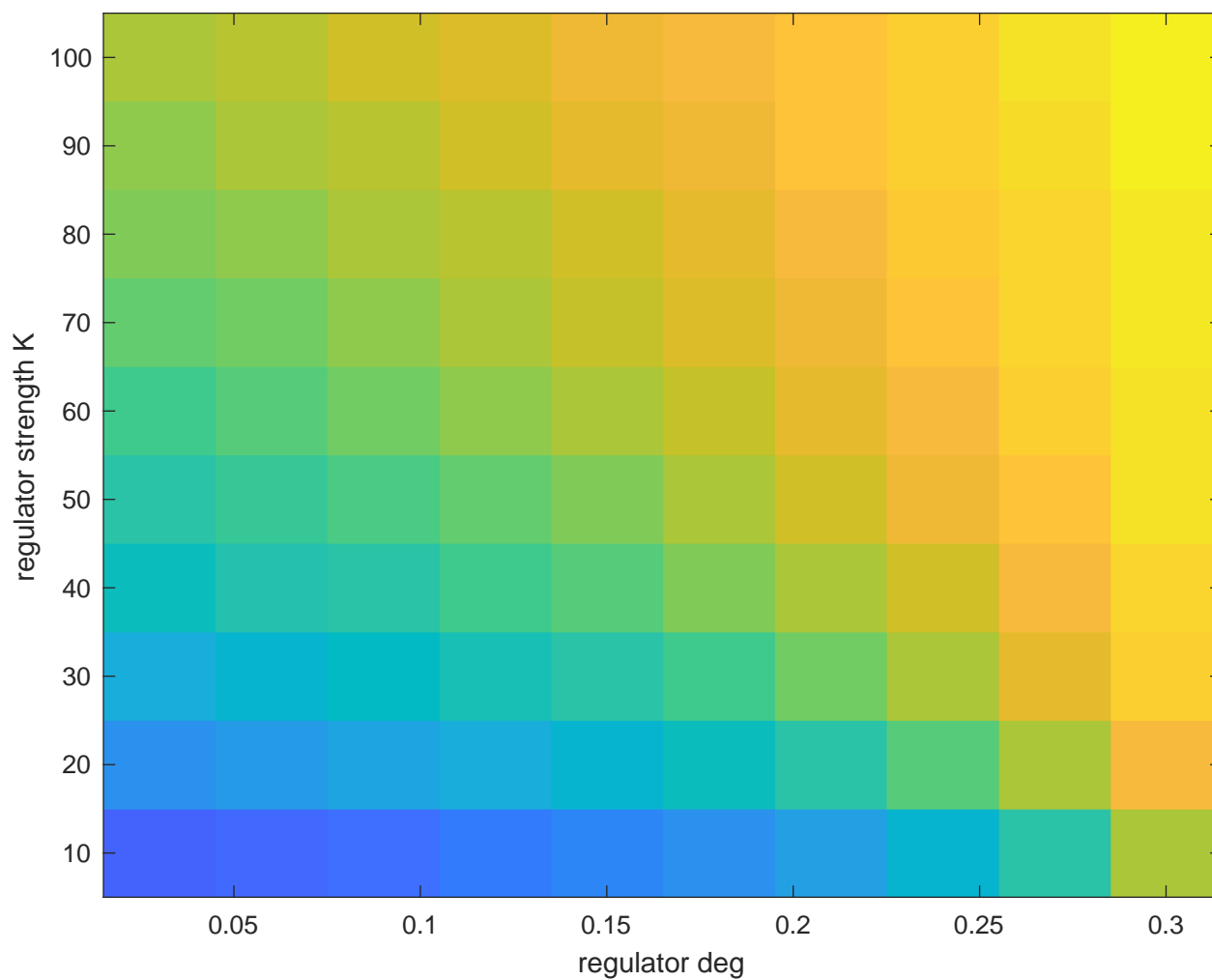

Supplement: Supplementary file 4 — Source Data [file 41467_2022_33058_MOESM4_ESM.zip › Source_Data_and_Source_Code_Final_Revision/Figure_1&Supplementary_FigureS3/Simulation-FIGURE_1/heatmap_impls_mag-2.pdf]

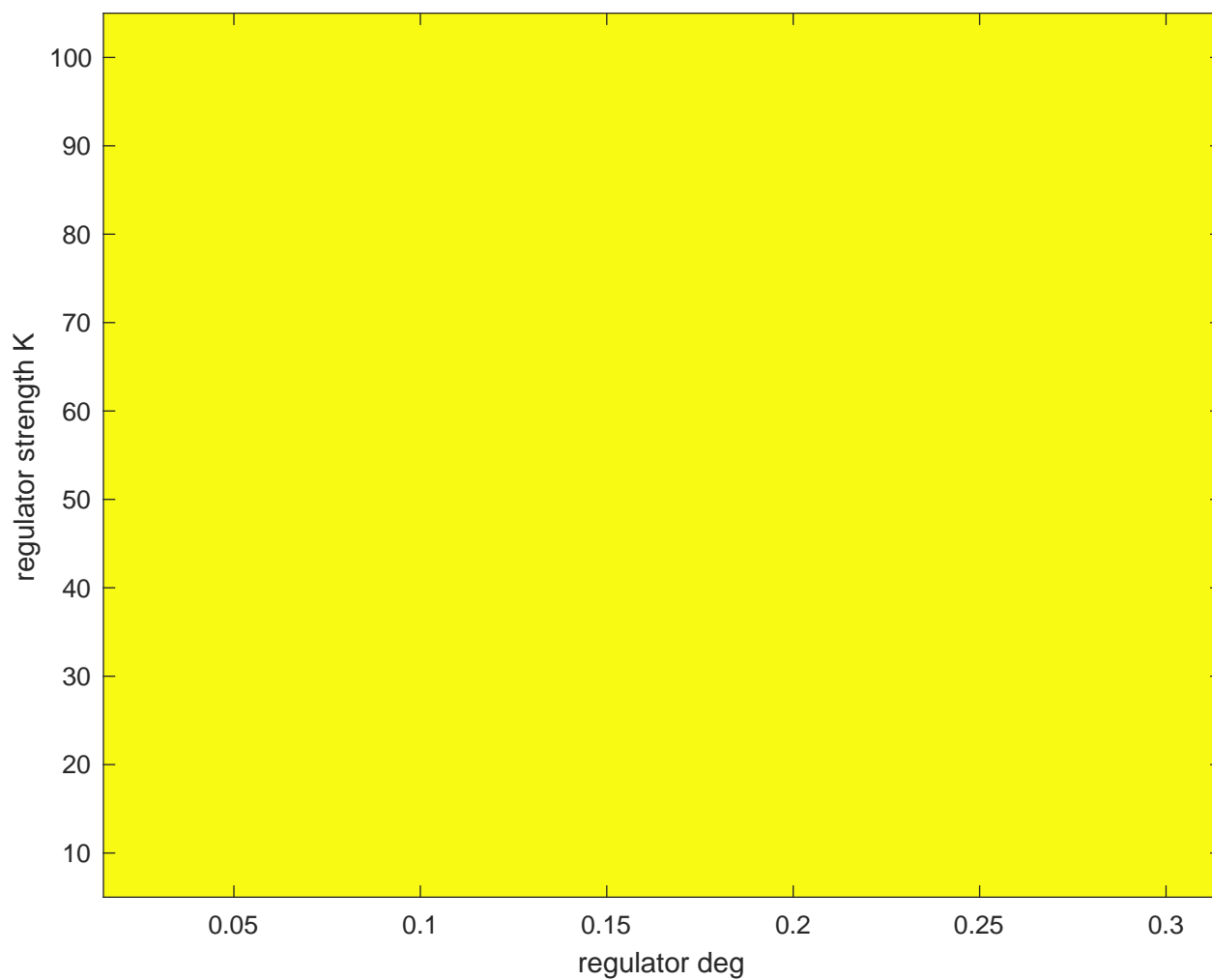

Supplement: Supplementary file 4 — Source Data [file 41467_2022_33058_MOESM4_ESM.zip › Source_Data_and_Source_Code_Final_Revision/Figure_1&Supplementary_FigureS3/Simulation-FIGURE_1/heatmap_impls_mag-1.pdf]

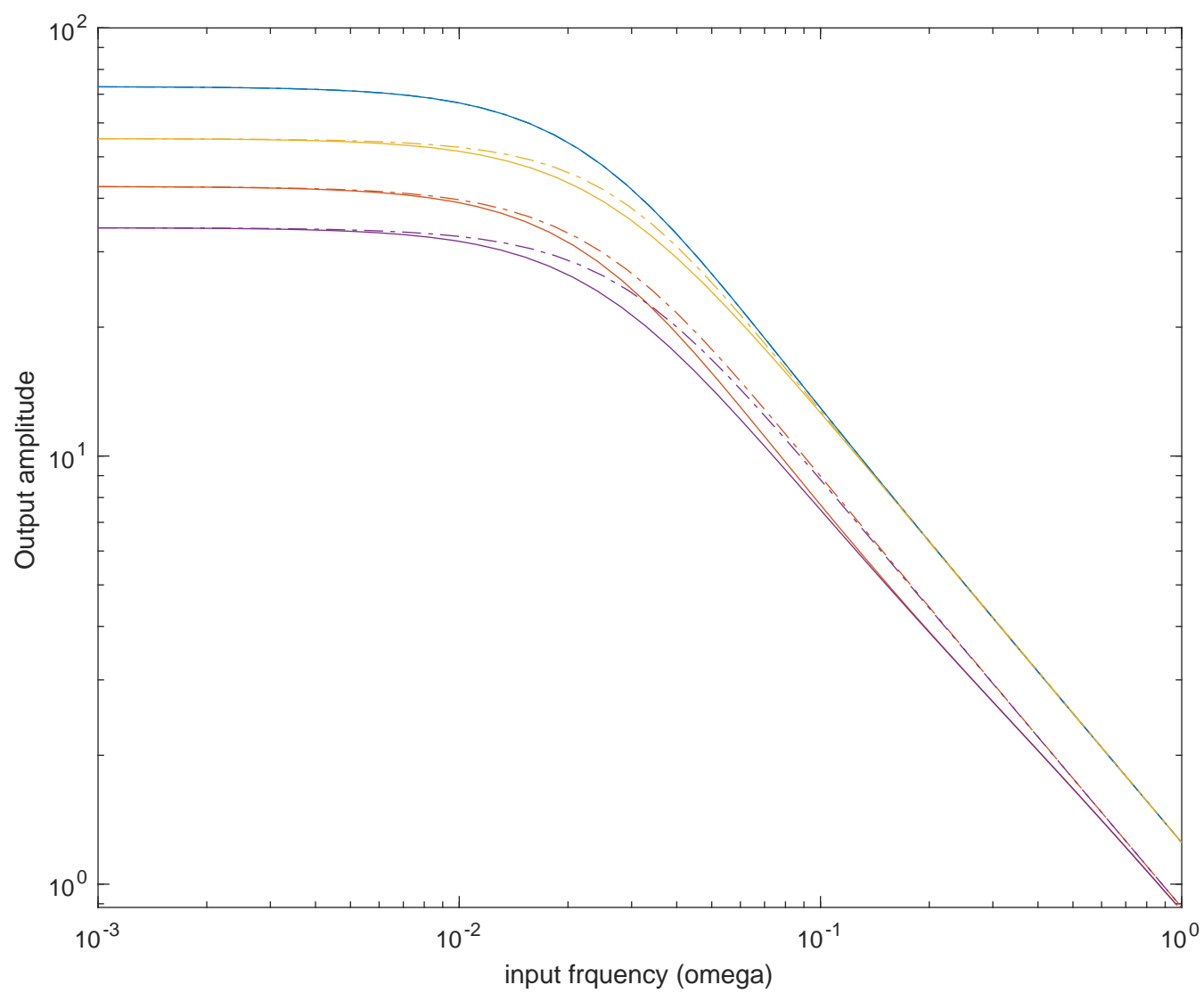

Supplement: Supplementary file 4 — Source Data [file 41467_2022_33058_MOESM4_ESM.zip › Source_Data_and_Source_Code_Final_Revision/Figure_1&Supplementary_FigureS3/Simulation-FIGURE_1/bode_mag_RNAvsProtein_loglog.pdf]

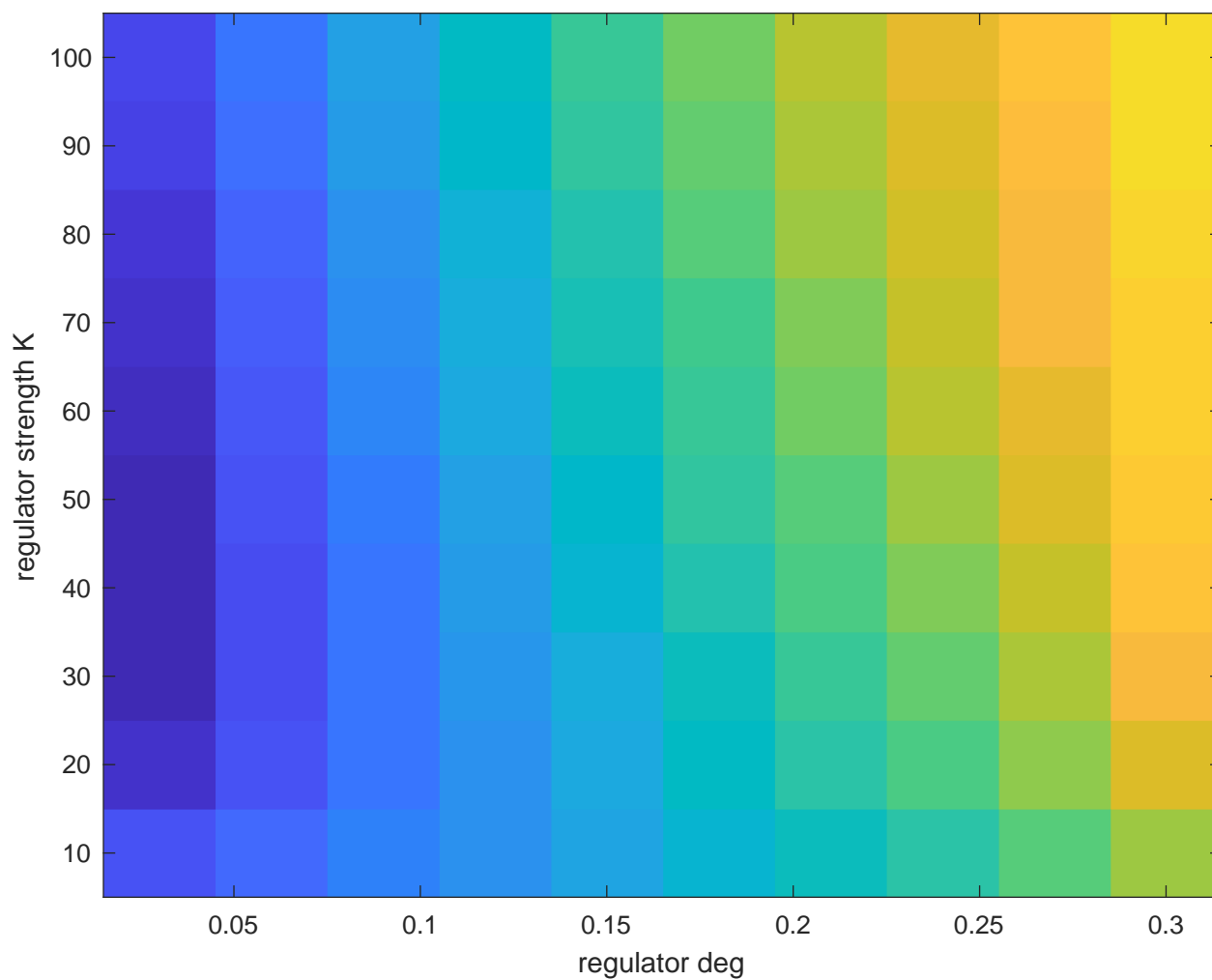

Supplement: Supplementary file 4 — Source Data [file 41467_2022_33058_MOESM4_ESM.zip › Source_Data_and_Source_Code_Final_Revision/Figure_1&Supplementary_FigureS3/Simulation-FIGURE_1/heatmap_step_settime-4.pdf]

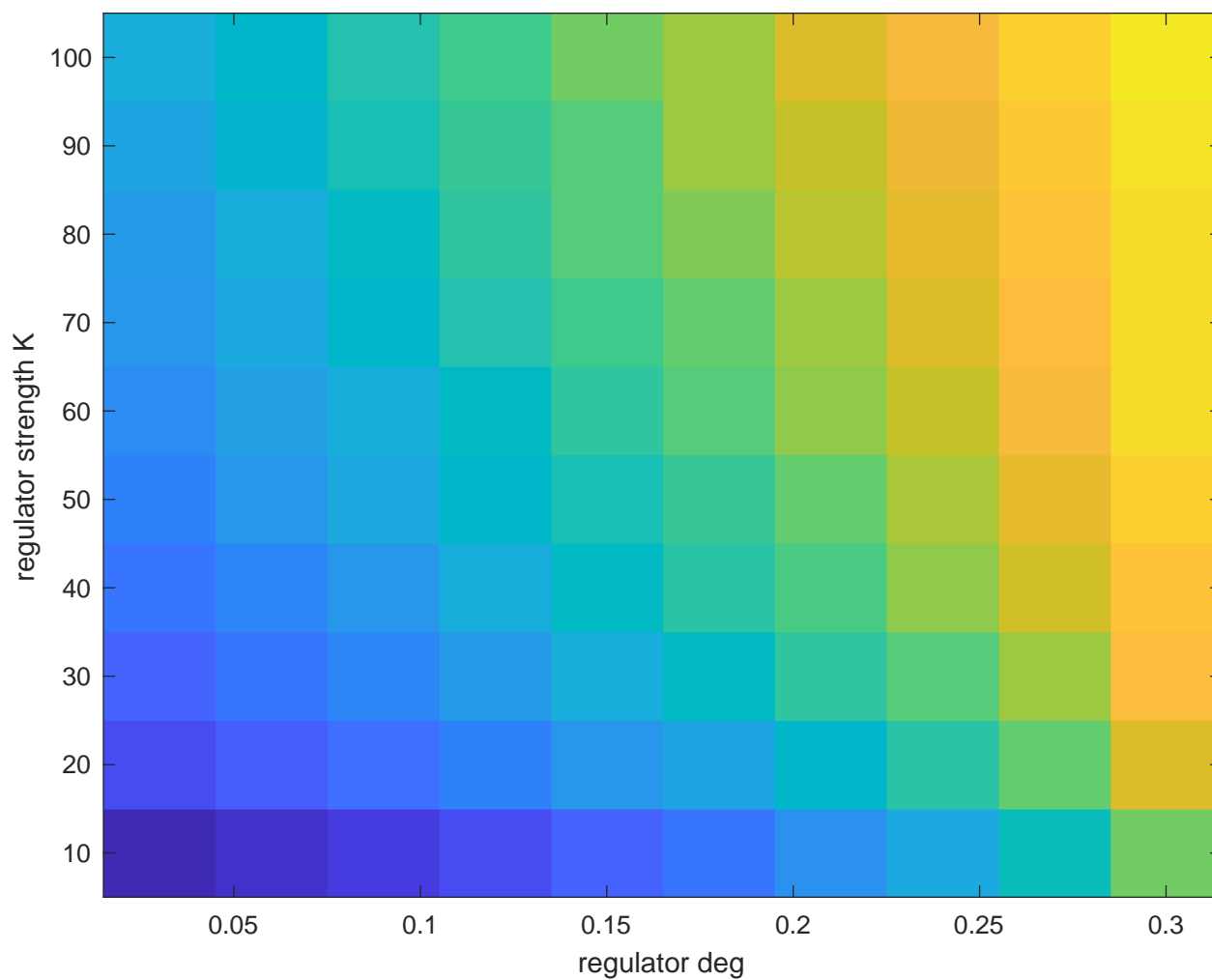

Supplement: Supplementary file 4 — Source Data [file 41467_2022_33058_MOESM4_ESM.zip › Source_Data_and_Source_Code_Final_Revision/Figure_1&Supplementary_FigureS3/Simulation-FIGURE_1/heatmap_impls_settime-4.pdf]

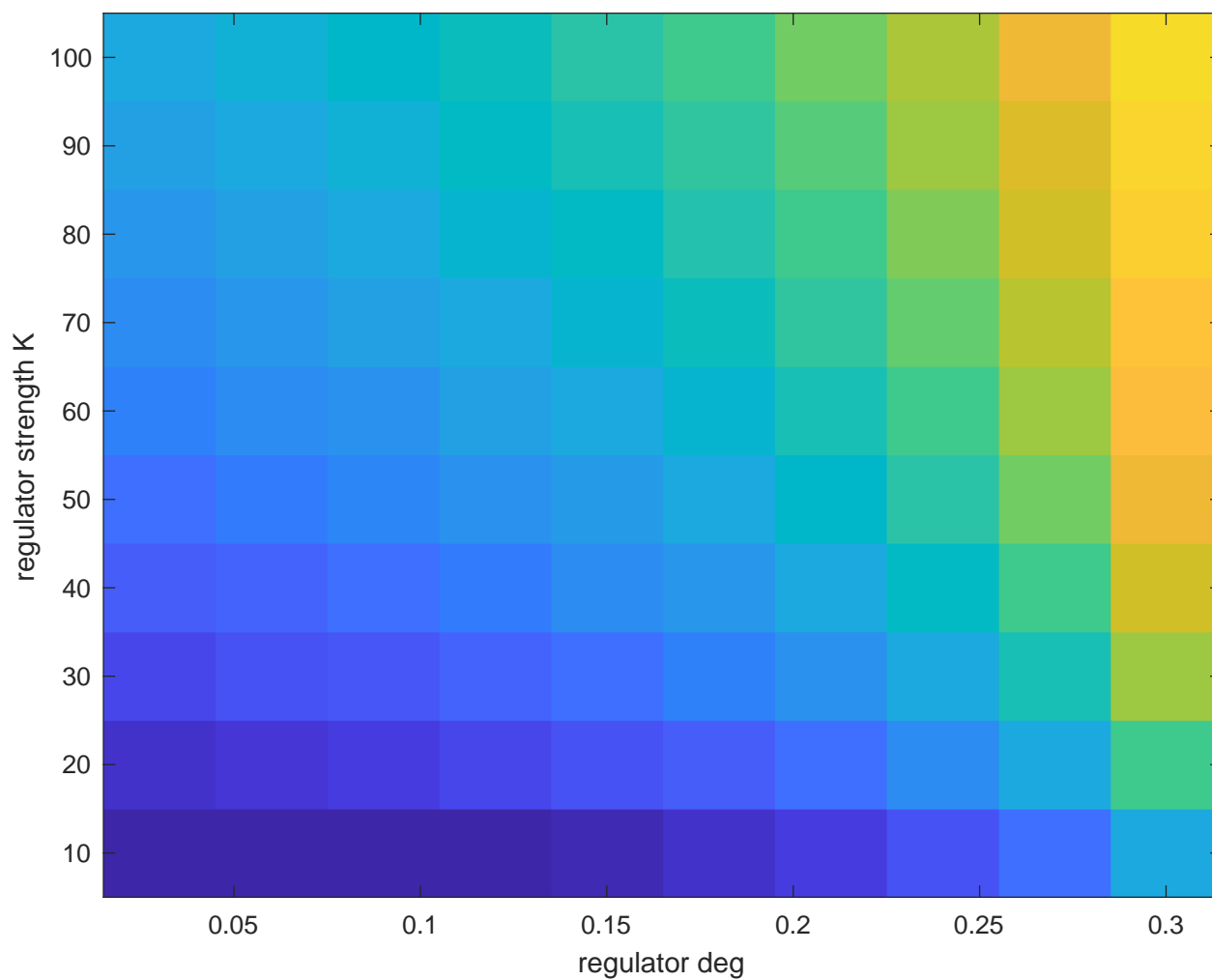

Supplement: Supplementary file 4 — Source Data [file 41467_2022_33058_MOESM4_ESM.zip › Source_Data_and_Source_Code_Final_Revision/Figure_1&Supplementary_FigureS3/Simulation-FIGURE_1/heatmap_step_mag-4.pdf]

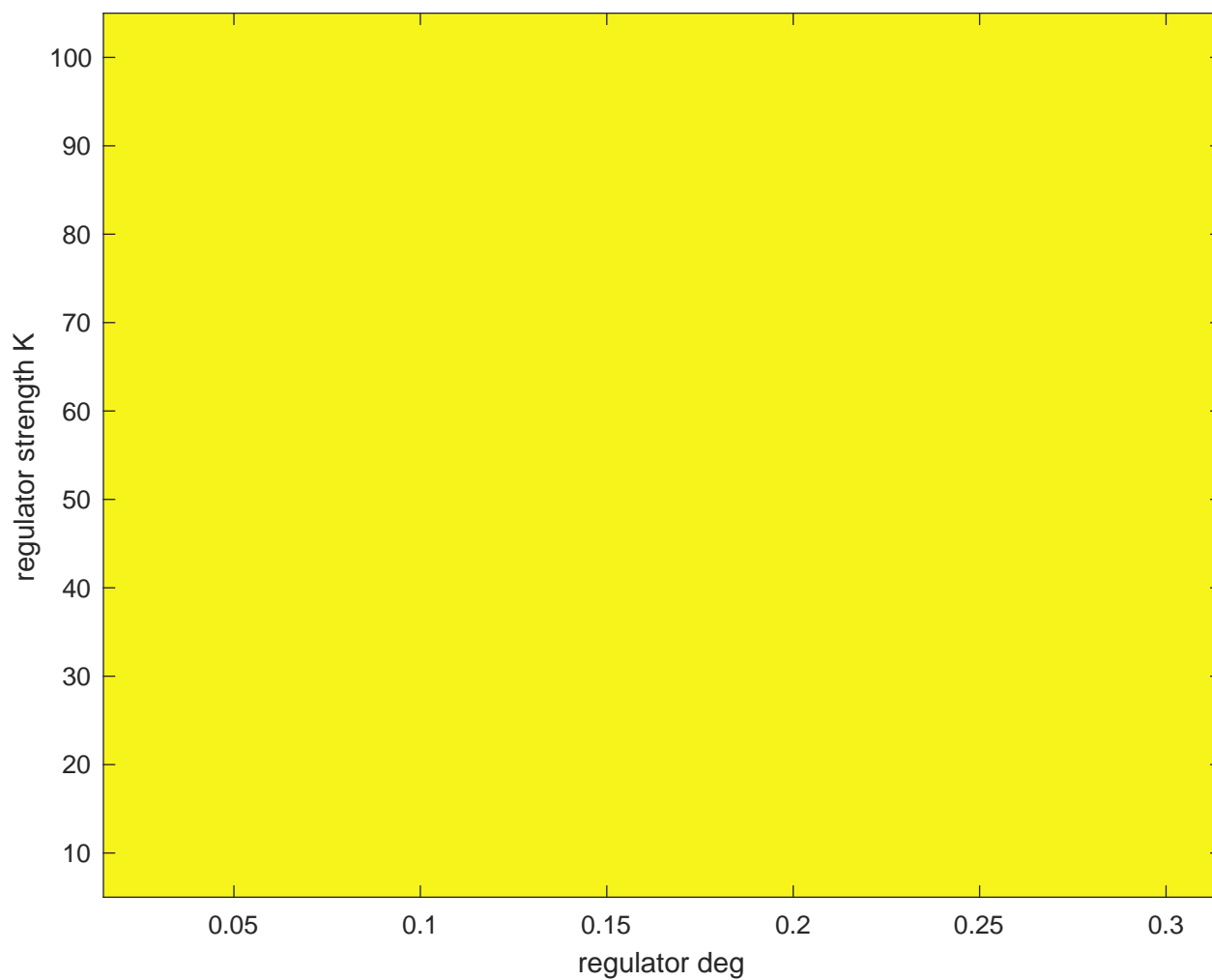

Supplement: Supplementary file 4 — Source Data [file 41467_2022_33058_MOESM4_ESM.zip › Source_Data_and_Source_Code_Final_Revision/Figure_1&Supplementary_FigureS3/Simulation-FIGURE_1/heatmap_step_settime-1.pdf]

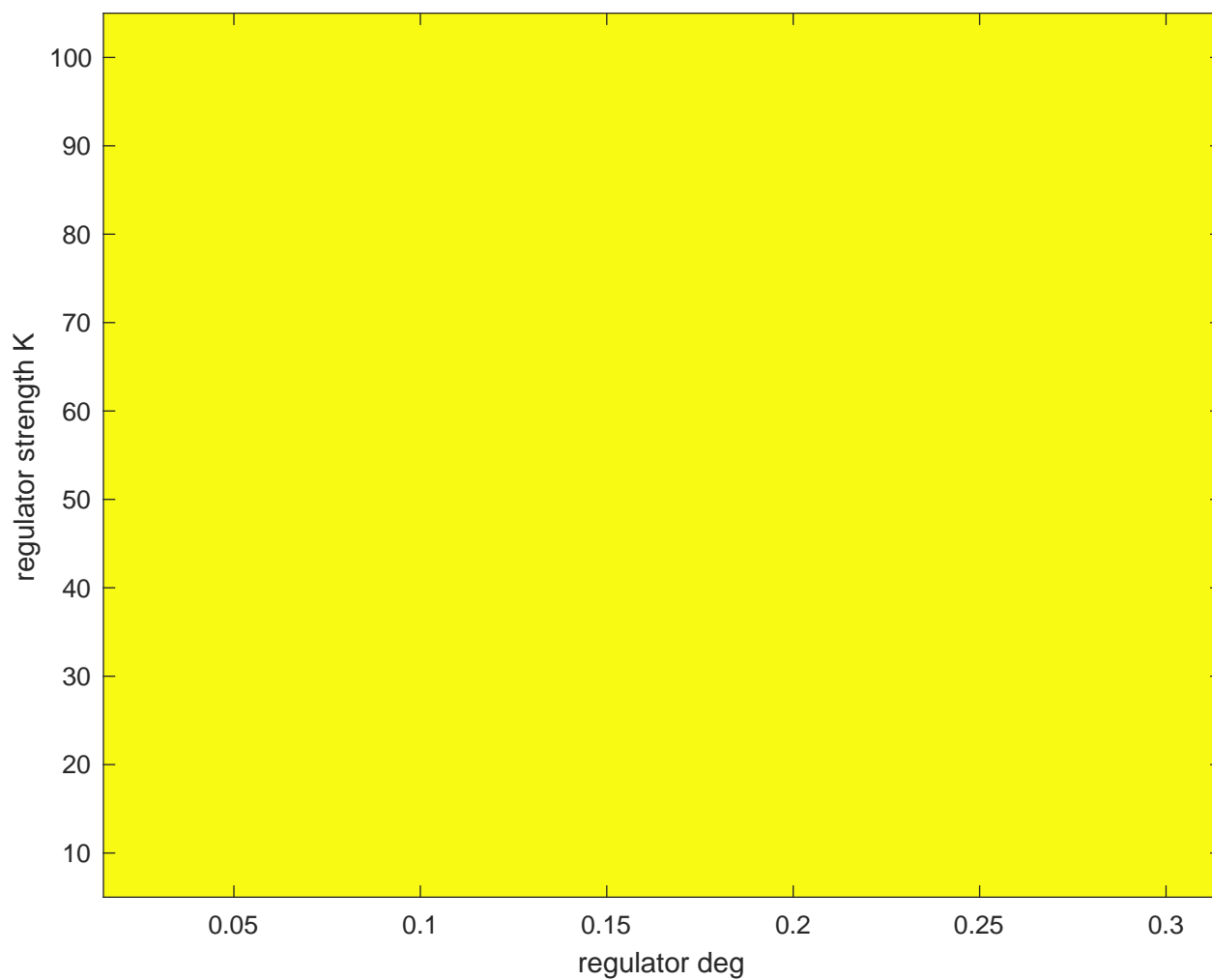

Supplement: Supplementary file 4 — Source Data [file 41467_2022_33058_MOESM4_ESM.zip › Source_Data_and_Source_Code_Final_Revision/Figure_1&Supplementary_FigureS3/Simulation-FIGURE_1/heatmap_impls_settime-1.pdf]

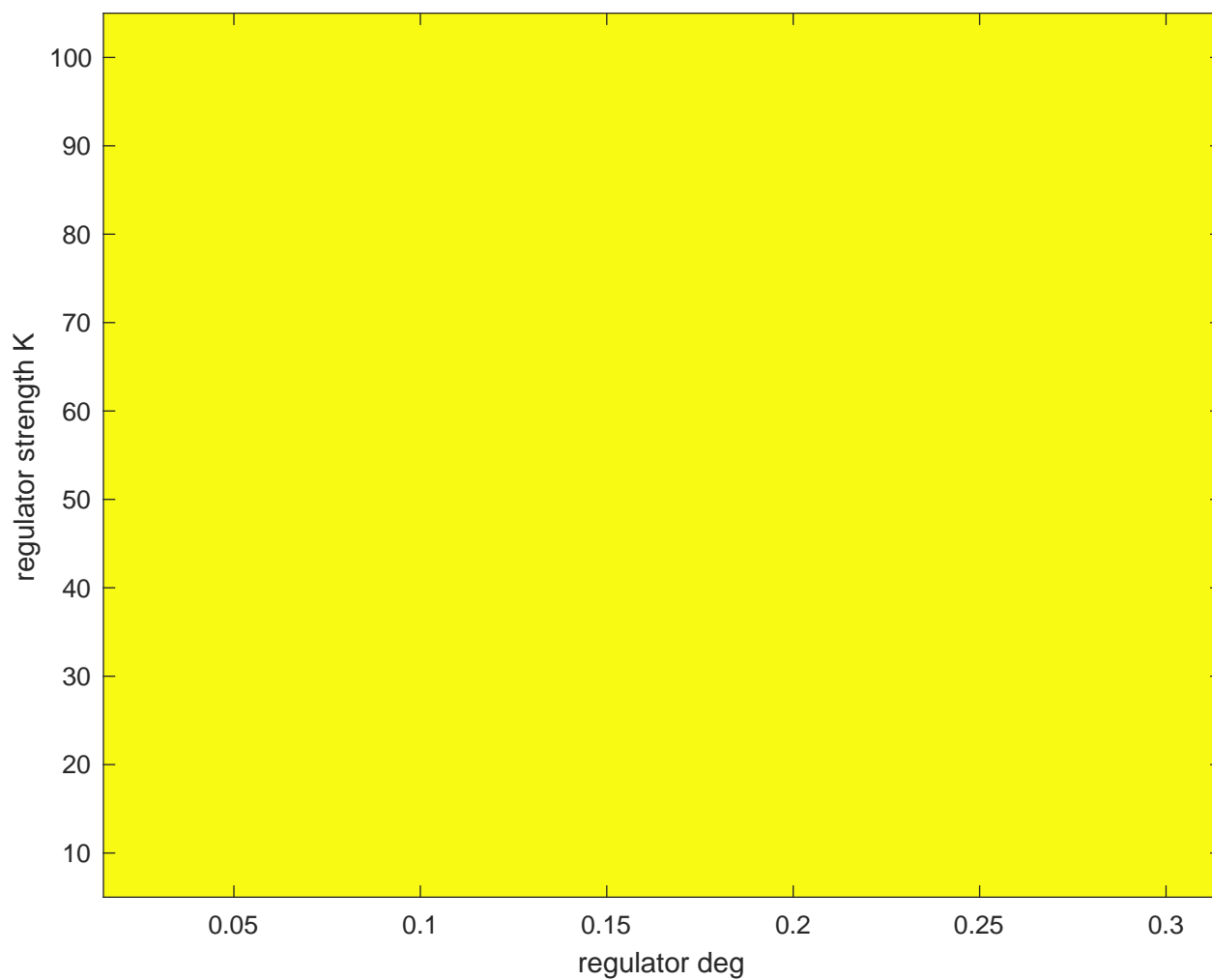

Supplement: Supplementary file 4 — Source Data [file 41467_2022_33058_MOESM4_ESM.zip › Source_Data_and_Source_Code_Final_Revision/Figure_1&Supplementary_FigureS3/Simulation-FIGURE_1/heatmap_step_mag-1.pdf]

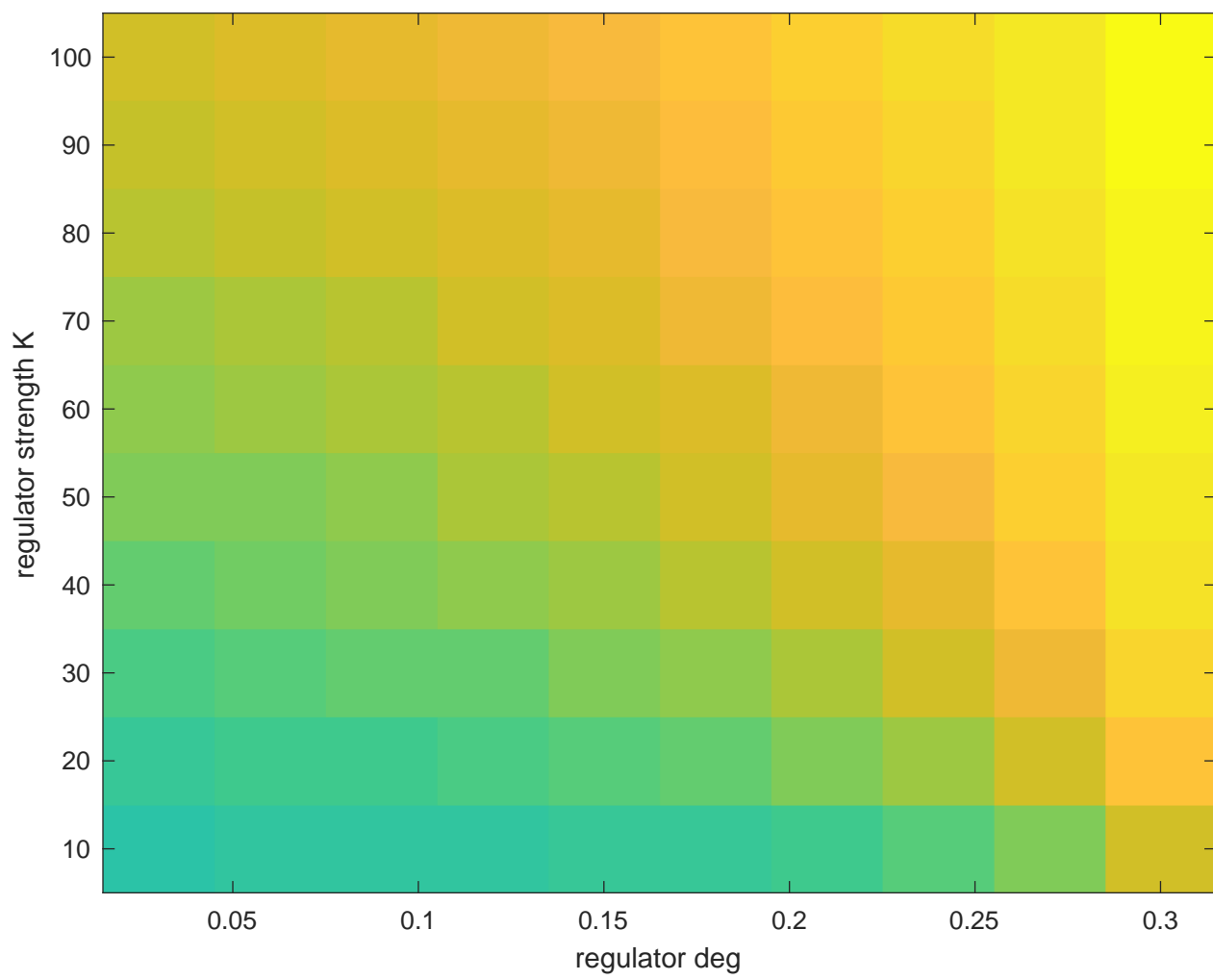

Supplement: Supplementary file 4 — Source Data [file 41467_2022_33058_MOESM4_ESM.zip › Source_Data_and_Source_Code_Final_Revision/Figure_1&Supplementary_FigureS3/Simulation-FIGURE_1/heatmap_step_mag-3.pdf]

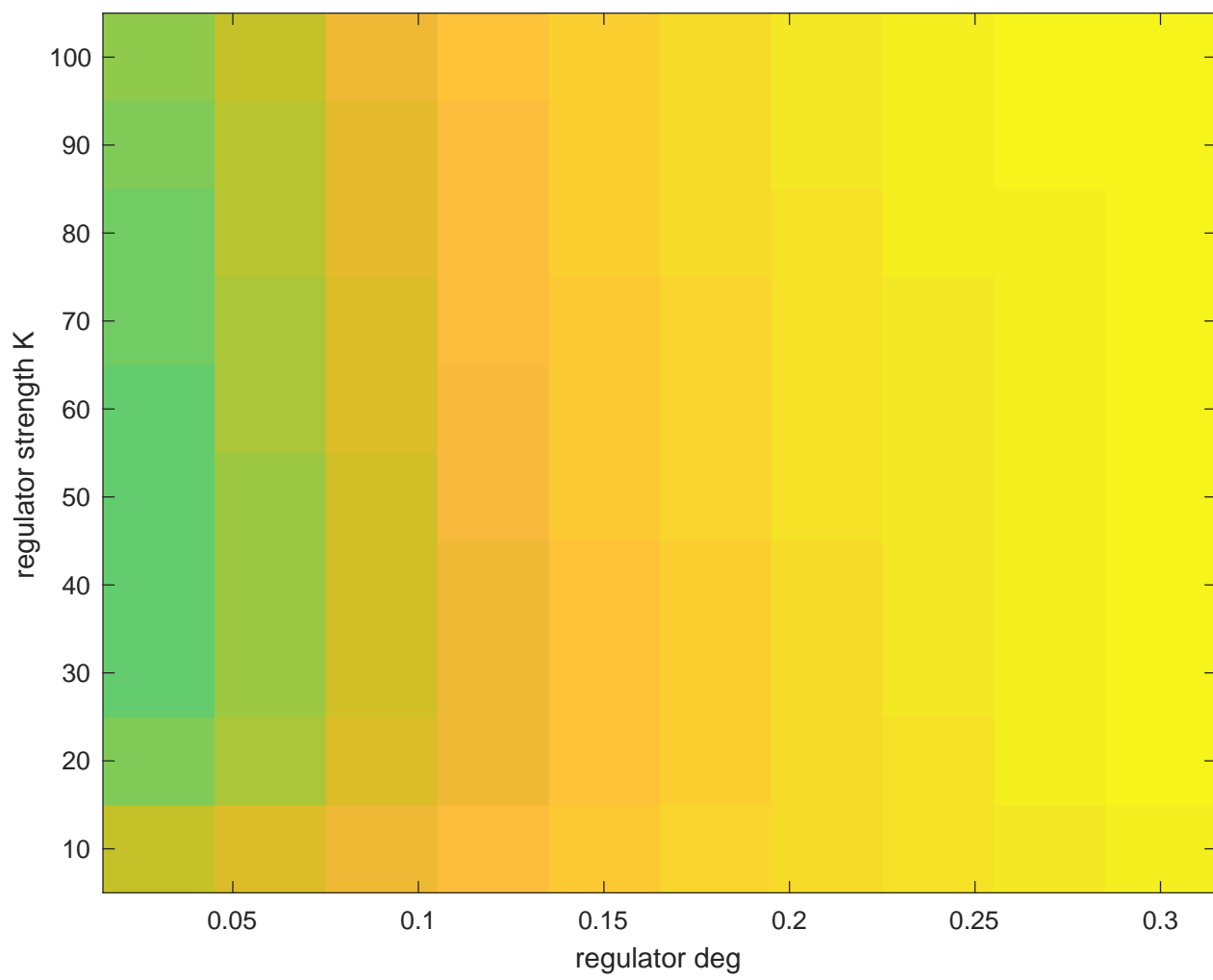

Supplement: Supplementary file 4 — Source Data [file 41467_2022_33058_MOESM4_ESM.zip › Source_Data_and_Source_Code_Final_Revision/Figure_1&Supplementary_FigureS3/Simulation-FIGURE_1/heatmap_step_settime-2.pdf]

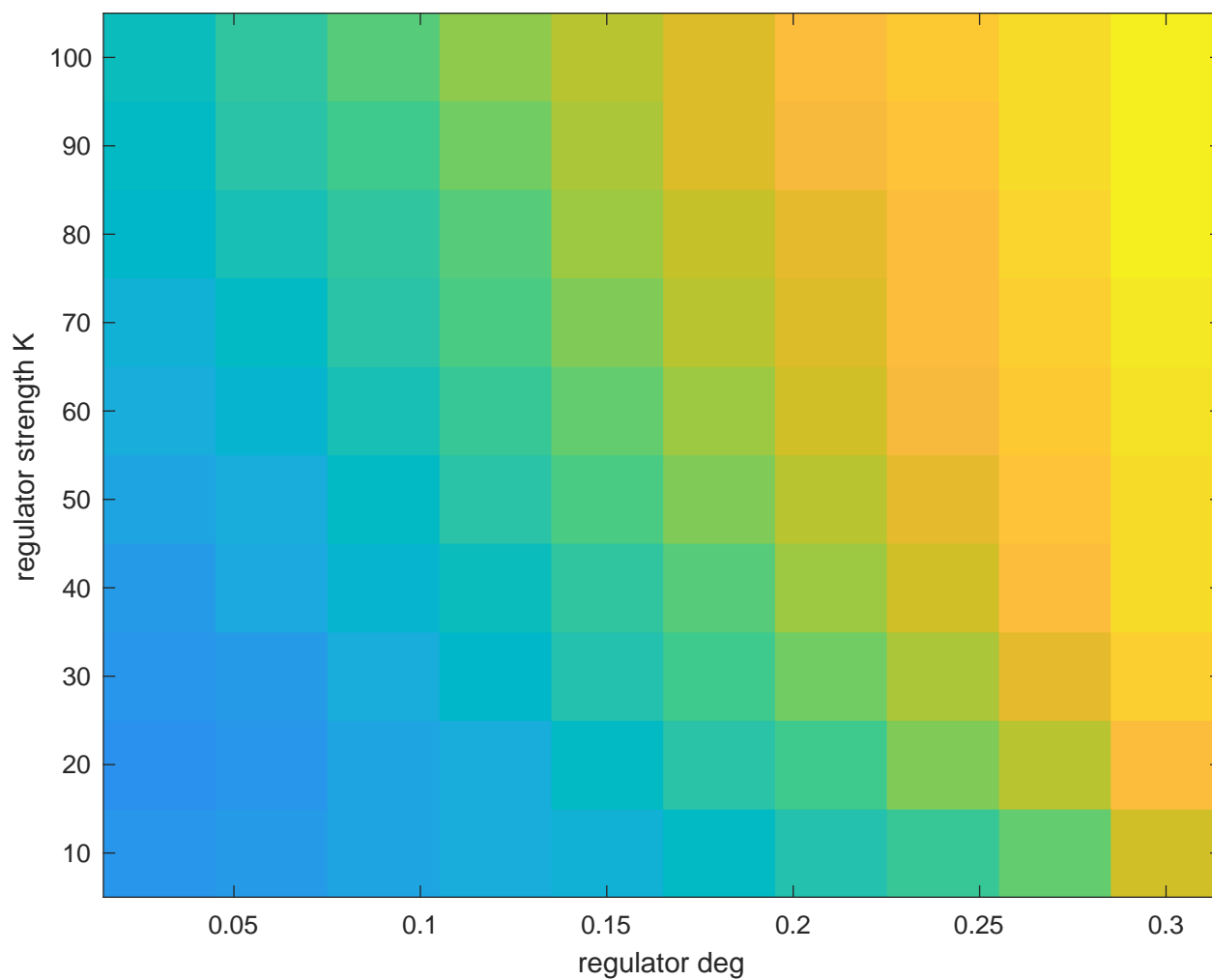

Supplement: Supplementary file 4 — Source Data [file 41467_2022_33058_MOESM4_ESM.zip › Source_Data_and_Source_Code_Final_Revision/Figure_1&Supplementary_FigureS3/Simulation-FIGURE_1/heatmap_impls_settime-3.pdf]

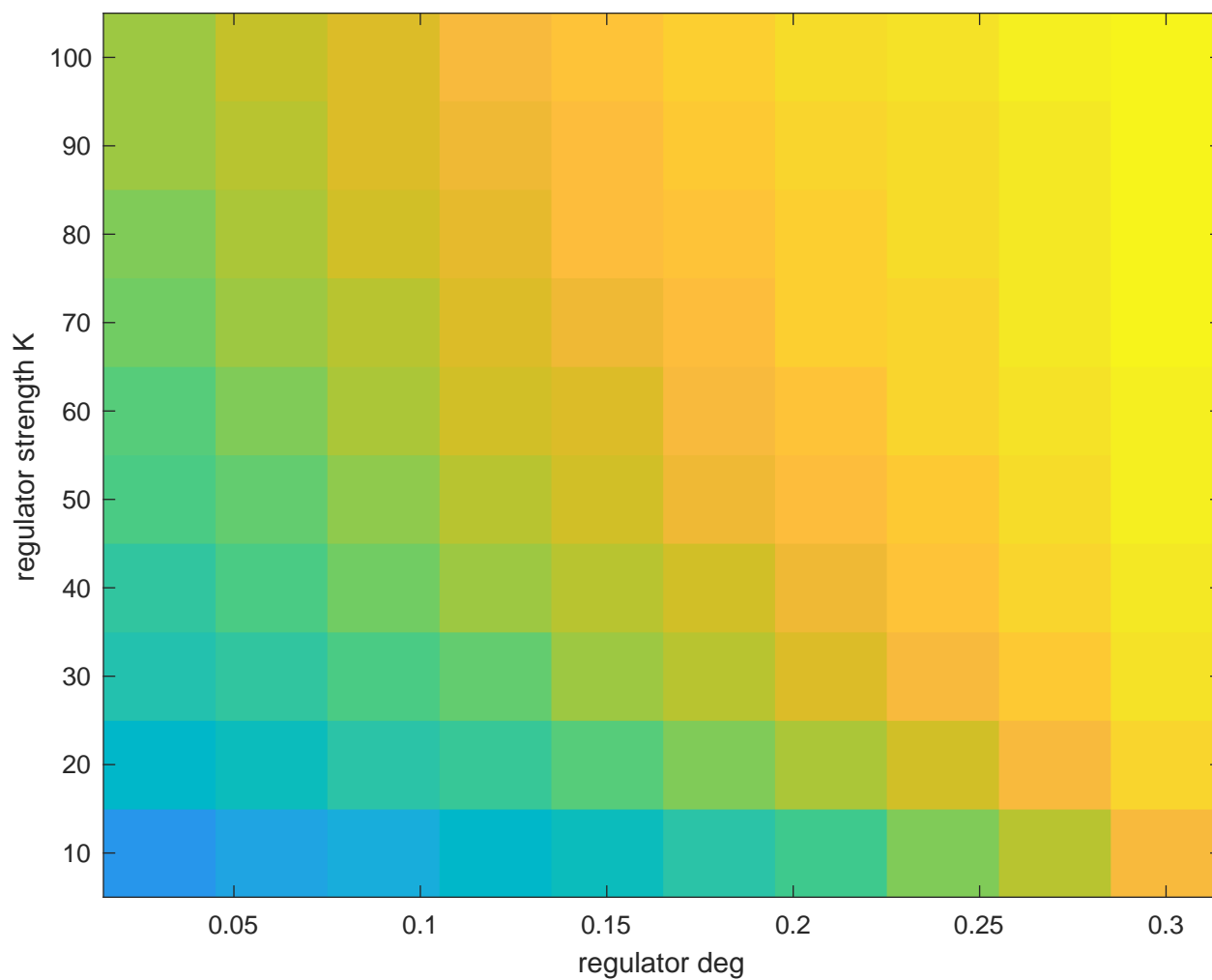

Supplement: Supplementary file 4 — Source Data [file 41467_2022_33058_MOESM4_ESM.zip › Source_Data_and_Source_Code_Final_Revision/Figure_1&Supplementary_FigureS3/Simulation-FIGURE_1/heatmap_impls_settime-2.pdf]

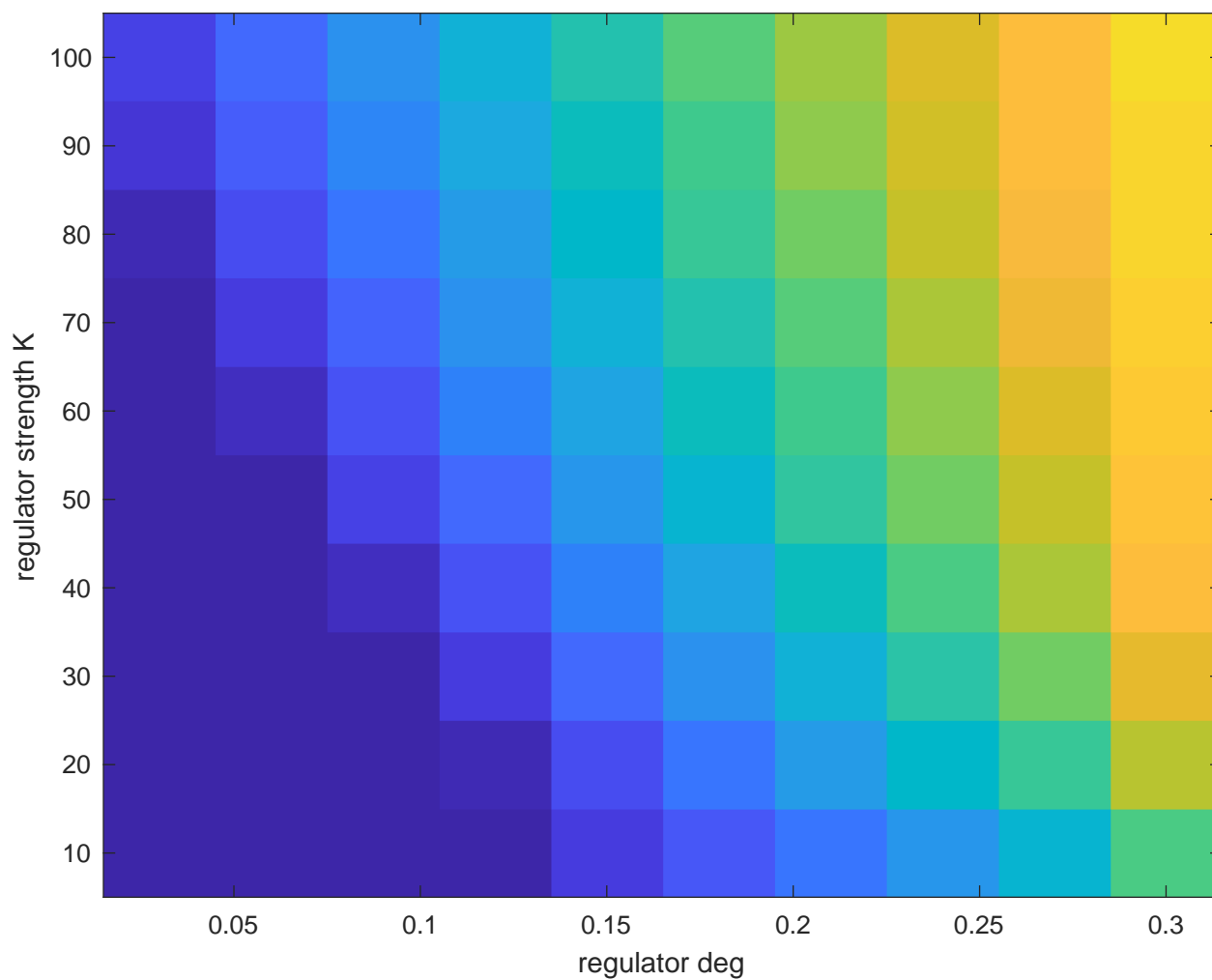

Supplement: Supplementary file 4 — Source Data [file 41467_2022_33058_MOESM4_ESM.zip › Source_Data_and_Source_Code_Final_Revision/Figure_1&Supplementary_FigureS3/Simulation-FIGURE_1/heatmap_step_settime-3.pdf]

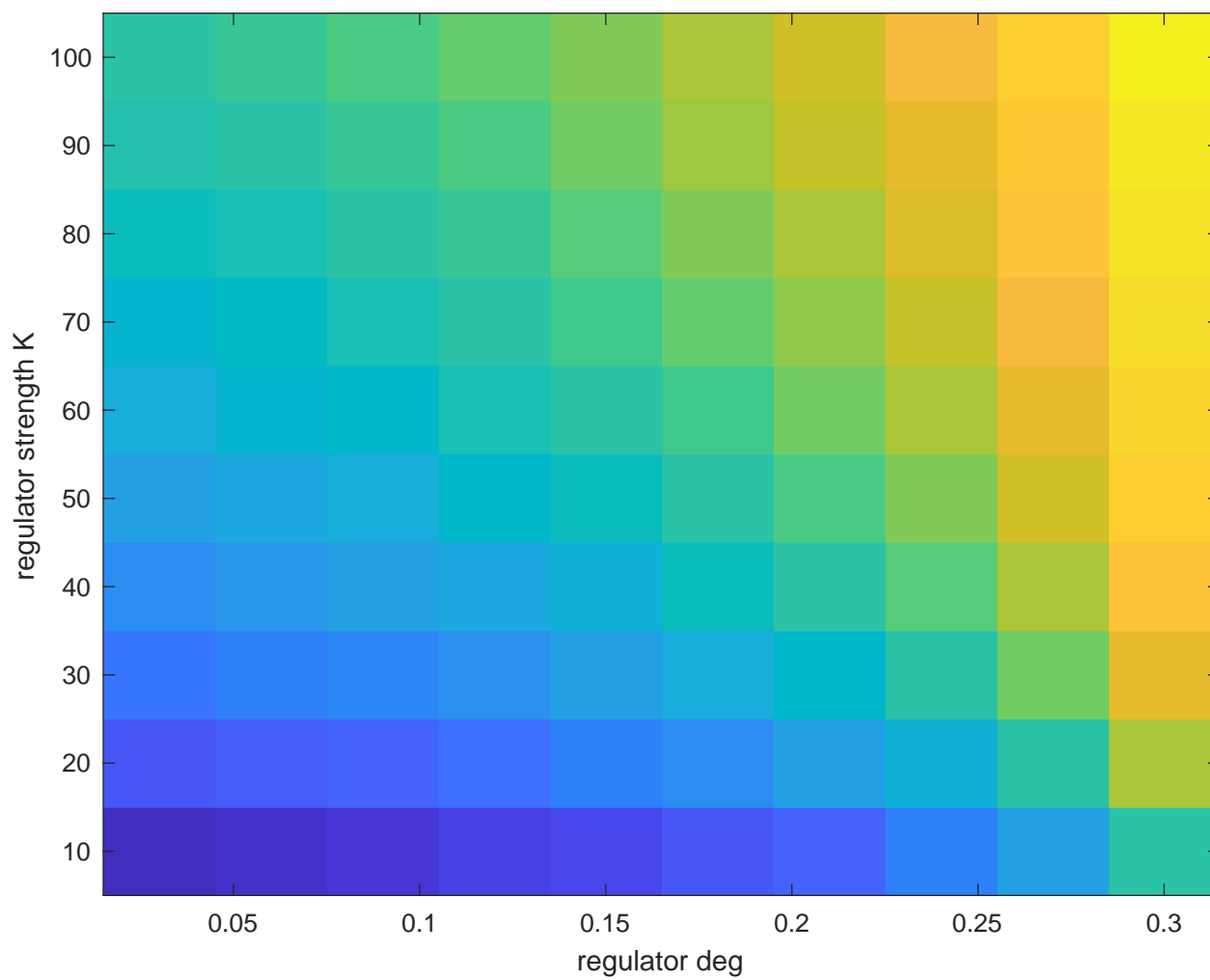

Supplement: Supplementary file 4 — Source Data [file 41467_2022_33058_MOESM4_ESM.zip › Source_Data_and_Source_Code_Final_Revision/Figure_1&Supplementary_FigureS3/Simulation-FIGURE_1/heatmap_step_mag-2.pdf]

FL/OD (AU)

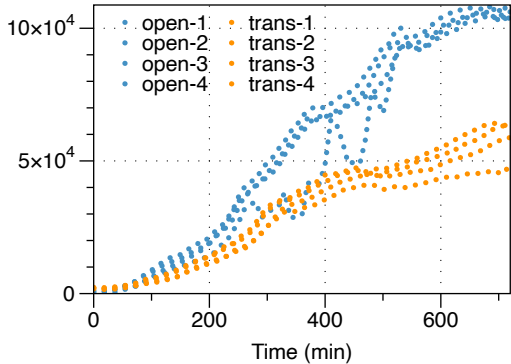

Supplement: Supplementary file 4 — Source Data [file 41467_2022_33058_MOESM4_ESM.zip › Source_Data_and_Source_Code_Final_Revision/Figure_3&Supplementary_FigureS1/Figure_3F/6.004R1.pdf]

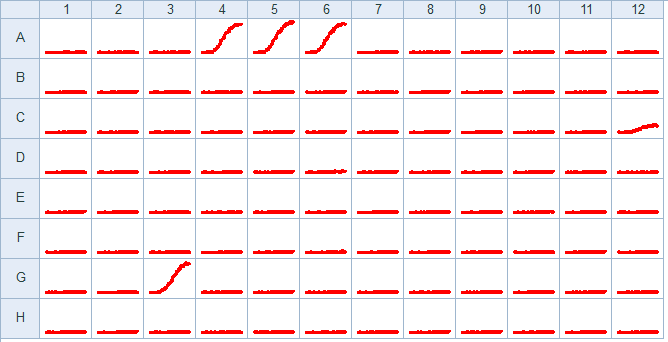

Supplement: Supplementary file 4 — Source Data [file 41467_2022_33058_MOESM4_ESM.zip › Source_Data_and_Source_Code_Final_Revision/Figure_3&Supplementary_FigureS1/Supplementary_FigureS1/6.003R2-BLOCK2-capture.PNG]

FL/OD

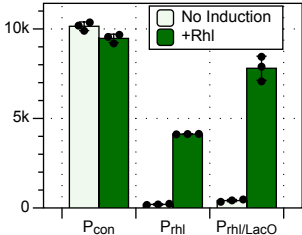

Supplement: Supplementary file 4 — Source Data [file 41467_2022_33058_MOESM4_ESM.zip › Source_Data_and_Source_Code_Final_Revision/Figure_3&Supplementary_FigureS1/Figure_3B/1.005R1 all pionts.pdf]

FL/OD

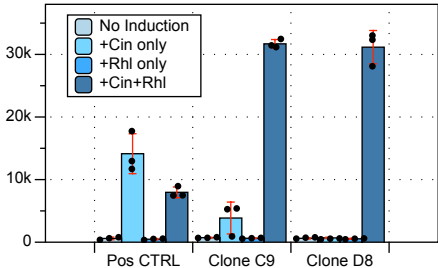

Supplement: Supplementary file 4 — Source Data [file 41467_2022_33058_MOESM4_ESM.zip › Source_Data_and_Source_Code_Final_Revision/Figure_3&Supplementary_FigureS1/Figure_3E/2.012R1-all pionts.pdf]

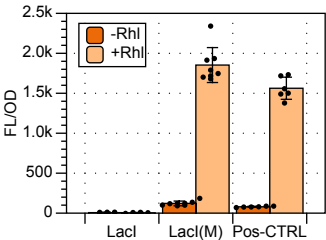

Supplement: Supplementary file 4 — Source Data [file 41467_2022_33058_MOESM4_ESM.zip › Source_Data_and_Source_Code_Final_Revision/Figure_3&Supplementary_FigureS1/Figure_3D/3.005R2-all pionts.pdf]
