# Supplementary material for: Layered feedback control overcomes performance trade-off in synthetic biomolecular networks
Source: Nat Commun. 2022 Sep 14;13:5393. doi: 10.1038/s41467-022-33058-6 (PMC9474519; doi:10.1038/s41467-022-33058-6)
Supplement: Supplementary file 4 — Source Data [file 41467_2022_33058_MOESM4_ESM.zip › Source_Data_and_Source_Code_Final_Revision/Figure_6&Supplementary_FigureS6-S8/A11.02R39-Temp2directions-24X/30C/Note.rtf]

After data exclusion, there are 19 ctrl, 24 trans, 19 cis and 22 layered in the data
Disturbance ranges from 380-500
