# Supplementary material for: Layered feedback control overcomes performance trade-off in synthetic biomolecular networks
Source: Nat Commun. 2022 Sep 14;13:5393. doi: 10.1038/s41467-022-33058-6 (PMC9474519; doi:10.1038/s41467-022-33058-6)
Supplement: Supplementary file 4 — Source Data [file 41467_2022_33058_MOESM4_ESM.zip › Source_Data_and_Source_Code_Final_Revision/Figure_6&Supplementary_FigureS6-S8/A11.02R42&43-Glucose2directions-24X/A11.02R42-Glucose-dip-24X/scaled_disturb1.2x1.5.pdf]

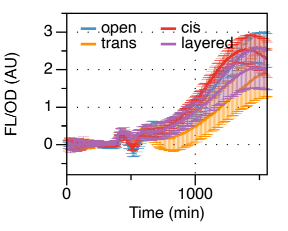
open
cis
trans
layered
FL/OD (AU)
3
2
1
0
0
1000
Time (min)
