# Supplementary material for: Layered feedback control overcomes performance trade-off in synthetic biomolecular networks
Source: Nat Commun. 2022 Sep 14;13:5393. doi: 10.1038/s41467-022-33058-6 (PMC9474519; doi:10.1038/s41467-022-33058-6)
Supplement: Supplementary file 4 — Source Data [file 41467_2022_33058_MOESM4_ESM.zip › Source_Data_and_Source_Code_Final_Revision/Figure_6&Supplementary_FigureS6-S8/A11.02R36-AHLspike-24X/Tx_spike_model/Simulation-RcisPtrans_tx_spike/trade-off-tx-spike.pdf]

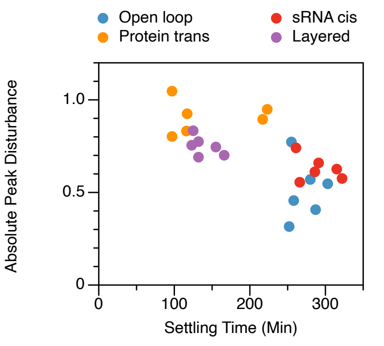

Open loop
sRNA cis
Protein trans
Layered
Absolute Peak Disturbance
1.0
0.5
0
0
100
200
300
Settling Time (Min)
