# Supplementary material for: Layered feedback control overcomes performance trade-off in synthetic biomolecular networks
Source: Nat Commun. 2022 Sep 14;13:5393. doi: 10.1038/s41467-022-33058-6 (PMC9474519; doi:10.1038/s41467-022-33058-6)
Supplement: Supplementary file 4 — Source Data [file 41467_2022_33058_MOESM4_ESM.zip › Source_Data_and_Source_Code_Final_Revision/Figure_6&Supplementary_FigureS6-S8/A11.02R39-Temp2directions-24X/Temp_disturb_model/Simulation-RcisPtrans-withRNAfolding/tempto42C.pdf]

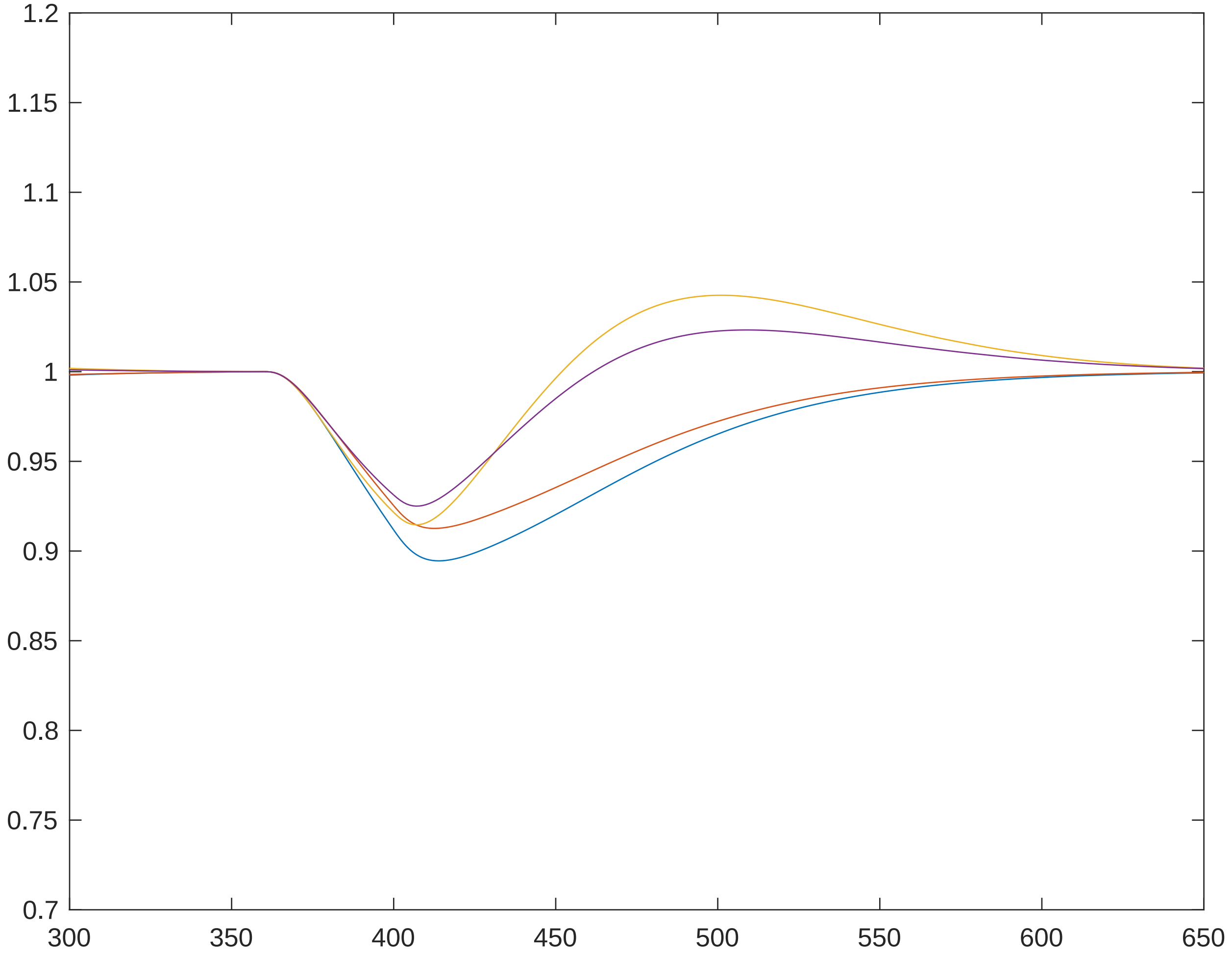
1.2
1.15
1.1
1.05
1
0.95
0.9
0.85
0.8
0.75
0.7
300
350
400
450
500
550
600
650
